# Bring The Noise: Three Rules for Improving Thermospheric Density Retrieval From LEO POD Data

**DOI:** 10.1007/s40295-026-00636-5

**Published:** 2026-10-22

**Authors:** Charles Constant, Shaylah M. Mutschler, Santosh Bhattarai, Marcin Pilinski

**Affiliations:** 1 University College London, London, UK; 2 Space Environment Technologies, Pacific Palisades, USA; 3 University of Colorado Boulder, Laboratory for Atmospheric and Space Physics, Boulder, USA

**Keywords:** Thermospheric mass density retrieval, Precise orbit determination, Energy dissipation rate, Low earth orbit, Data assimilation

## Abstract

Thermospheric mass density knowledge remains a primary driver of operational uncertainty for Low Earth Orbit operations, yet current sources of density observations suffer from limited latency, accuracy, and/or licensing issues. The growing body of Precise Orbit Determination data from emerging Low Earth Orbit constellations offers a promising source of thermospheric mass density measurements. Realising this potential requires the application of methods which can convert Precise Orbit Determination data into reliable density observations. This work provides an assessment of the Energy Dissipation Rate technique. Using a simulated constellation of up to eighteen Starlink-like satellites, we quantify the relationship between the quality of retrieved mass densities and four major influencing factors: Precise Orbit Determination noise level, drag acceleration magnitude, estimation fit-span, and the number of co-orbital spacecraft whose independent density estimates are averaged. Densities retrieved via the Energy Dissipation Rate method are also compared with densities that have been assimilated into a background density model. We identify conditions under which non-assimilated retrievals can outperform their assimilated counterparts, demonstrating the benefits of averaging data from multiple satellites along the same orbital track. Our results reveal three rules of thumb that describe how retrieval error scales as a function of fit-span, satellite count, and drag level. Doubling the fit-span roughly halves the RMS percentage error; adding co-orbital satellites reduces error as ${\sim }N^{-0.6}$ (unassimilated) or ${\sim }N^{-0.25}$ (assimilated); and drag-acceleration sensitivity follows a logistic response with the steepest gains between $2\times 10^{-8}$ and $2\times 10^{-7}\,\mathrm {m\,s^{-2}}$. Together, these findings provide the first systematic quantification of how density-retrieval errors scale with Precise Orbit Determination noise, drag-acceleration level, fit-span, and satellite count. The resulting analytical approximation captures over 89% of the variance in simulated retrieval errors and is intended to support the design of next-generation assimilative thermospheric mass density models.

## Introduction

The rapid expansion of Low Earth Orbit (LEO) satellite constellations over the past half-decade has fundamentally altered both the promise and the risk profile of the near-Earth space environment. With over ten thousand spacecraft already in operation and certain projections suggesting a further order-of-magnitude increase by the early 2030 s [2, 30], the risk of collisions and the attendant threat of a cascading Kessler-type event have escalated [39]. Effective risk mitigation depends heavily on a key operational component: accurate, high-cadence, nowcasting and forecasting specifications of neutral thermospheric mass density (hereafter density) [41, 53, 57, 71]. Atmospheric drag acceleration scales linearly with mass density; because mass and number density differ by a composition-dependent mean molecular mass, the distinction matters and “density” throughout this paper refers exclusively to neutral mass density.

At the same time, the proliferation of hardware in space presents an unprecedented data-gathering opportunity, one which offers the potential to offset the very risks these constellations pose. Each satellite in LEO can in principle serve as a moving sensor, indirectly sampling the thermosphere along its orbital path [28, 54, 62]. At the current scale of deployment, and with even greater numbers on the horizon, the satellite operations and space-weather communities stand to achieve a step change in empirical density modelling. A sufficiently dense network of LEO satellites could deliver the spatio-temporal coverage required to constrain the next generation of thermospheric density models.

Realising this opportunity, however, depends on the type of tracking data available. Operational satellites usually actively cooperate in the tracking process by releasing Precise Orbit Determination (POD) products, state vectors (position and velocity as a function of time), or other telemetry; we refer to these as cooperatively tracked objects. Others provide no such data, and their orbits must be reconstructed from external observations such as radar or optical measurements, most widely disseminated in the form of Two-Line Element sets (TLEs); these are uncooperatively tracked objects. Both categories enable thermospheric density estimation, but with differences in accuracy, latency, temporal resolution (accelerometer-derived densities are sampled at intervals of seconds, whereas orbit-decay methods yield estimates over one or more orbital revolutions), and ultimately operational value.

To enable the step-change in modelling promised by large constellations, the community needs a clear understanding of how the increasing pool of potential cooperative tracking data can be exploited, their respective limitations, and strategies to address them. Research in this direction is emerging, and the present paper aims to contribute by generating results directly relevant to the primary stakeholders in model ideation and development- namely assimilative model developers and satellite operators. Providing empirically derived rules of thumb on factors such as required POD quality, number of satellites, and the time span of observations is essential to inform sound decision-making in this domain.

### State of Thermospheric Density Modelling

At present, three broad thermospheric density model families serve the operations community:

1. **Data-driven climatology models (also called semi-empirical models; non-circulation / non-fluid-solving):** These are typically parametric in nature and attempt to represent the statistically most probable state of the thermosphere for a given set of inputs. They do not solve the governing conservation equations of the upper atmosphere, and as such cannot reproduce the thermosphere’s evolution as a coupled dynamical system. Models in the Mass Spectrometer and Incoherent Scatter Radar (MSIS) series (e.g. NRLMSISE-00, MSIS 2.0) underpin many operational workflows, and remain widely used due to their low computational cost, favourable usage licenses, and thorough characterization within the literature [7, 53, 60].
2. **First-principles General Circulation Models (GCMs; also called numerical models; circulation / fluid-solving):** By solving the coupled physical equations of the upper atmosphere (e.g. Navier–Stokes), General Circulation Models are capable of reproducing physical features that emerge from system dynamics, across a variety of spatial and temporal scales. Examples include terminator waves, post-storm over-cooling, and the equatorial temperature and wind anomaly. However, the computational burden of these models is substantial, and they remain prohibitive for many stakeholders (e.g. satellite operators). They are most often run by specialist space weather organisations (e.g. National Oceanic and Atmospheric Administration Space Weather Prediction Center (NOAA SWPC) and the Met Office Space Weather Operations Center(MOSWOC)). Machine-learning surrogates are in development [40] and may provide a valuable compromise between physical fidelity and efficiency, echoing recent successes in terrestrial weather modelling [8, 37].
3. **Assimilative models:** In terrestrial weather modelling, ensemble-variational methods (e.g. Ensemble Four-Dimensional Variational (4DVar)) applied to GCMs represent the gold standard [31]. For the thermosphere, no 4DVar system exists, but several ensemble Kalman filter [24] assimilation systems have been developed for GCMs. These models blend observations to tie a model (data-driven or first-principles) back to reality. The core premise is that, where observations are too sparse or erroneous, their combination with a high-quality model provides an answer closer to the truth. While this framework is the backbone of terrestrial numerical weather prediction, it is still in its infancy for the space weather community- the High Accuracy Satellite Drag Model (HASDM; operational within the U.S. Government) is perhaps the most well-known model of this variant, with alternatives being made operational such as the recent deployment of the Advanced ENsemble electron density Assimilation System (AENeAS) [22] at the MOSWOC. For neutral mass density specifically, assimilative approaches include ensemble Kalman filter frameworks applied to general circulation models [18, 43, 50], operational calibration systems such as the Thermosphere Ionosphere Data Assimilation (TIDA) system [15], and coupled thermosphere-ionosphere assimilation studies [26].

### Observation Bottleneck

The skill of any assimilative model is bounded, on one hand by the quality of the observations available and, on the other, by the quality of the underlying model. In practice, three attributes of the assimilated observations drive the operational performance of an assimilative scheme: the number of observations, their accuracy, and their latency. State-of-the-art systems such as HASDM rely on calibration data from a network of 80+ uncooperatively tracked satellites [9, 10]. While this approach provides best-in-class performance [7, 68], these observations are sparse in both space and time [20] relative to the potential pool of cooperative data streams that could be harnessed from current and planned LEO constellations.

This sparsity introduces several shortcomings. Firstly, the temporal coverage may be insufficient to resolve the rapid density enhancements and relaxations that occur during geomagnetic storms, where increases of nearly an order of magnitude can unfold within only a few hours [56, 76]. Second, the set of calibration objects occupies orbits that are not necessarily distributed across regions where satellite traffic is most heavily concentrated. As a result, the regions of the thermosphere where accurate mass density knowledge is most operationally critical may not be adequately sampled. By contrast, POD data have the potential to address these limitations: they offer higher-cadence measurements (POD solutions are typically available at intervals of 30–60 s, compared with TLE epoch spacings of order 8 h [20]), higher spatial resolution [27], and substantially lower positional uncertainty. Reported 3D RMS errors for commercial-grade POD solutions are on the order of 1–2 m [4], whereas TLE-derived positions carry errors commonly reported at the kilometre level [16]. These data are generated within the most densely populated orbital shells, and as the LEO satellite population increases this data stream may expand to tens or perhaps hundreds of thousands of potentially assimilable sources.

### Density Observation Sources

The oldest and most widely used density proxy stems from uncooperative tracking data. Perhaps the most ubiquitous example is the TLE set, in continuous operational use since the late 1950 s [17, 34, 59]. TLEs encode a satellite’s ballistic coefficient along with changes in the mean motion that are sensitive to drag acceleration and thermospheric mass density, providing an orbit-averaged measure of density. While globally available, the data are noisy, low-cadence, and integrated over longer time periods (typically hours to days), limiting their value for timely, high-resolution modelling.

In contrast, high-resolution accelerometer data from missions such as the CHAllenging Minisatellite Payload (CHAMP) and the Gravity Recovery and Climate Experiment Follow-On (GRACE-FO) have underpinned thermosphere research for more than two decades [42, 67]. These dedicated missions furnish high-accuracy, high-temporal-resolution (often ≤30 s) measurements of non-gravitational forces. Isolating the drag component requires assumptions about solar radiation pressure, Earth radiation pressure, and thermal re-radiation, but once these are accounted for the resulting density retrievals represent the gold standard for validation across the literature [44, 53, 67]. Operationally, however, the computationally intensive processing, the latency of data production (often days to weeks), and the very limited number of satellites restrict their utility for real-time model assimilation.

Between these extremes lies the use of POD solutions derived from on-board Global Navigation Satellite System (GNSS) receivers (satellite laser ranging (SLR) offers a comparable orbit-based route to density retrieval [75], though SLR infrastructure constraints limit its scalability). Over the past 10–15 years, this approach has been explored as an intermediate solution, offering higher cadence than uncooperative measurements and lower latency than accelerometer data [13, 27, 35, 63]. A key advantage is that POD-derived densities are effectively a data of opportunity: they exploit GNSS hardware already carried on board spacecraft for navigation, avoiding the cost and complexity of dedicated accelerometers. This results in lower mission hardware costs, while still enabling density retrievals at useful spatio-temporal scales (orbit-averaged estimates every one to a few orbital revolutions, i.e. roughly 90–300 min cadence, compared with ≤30 s for accelerometers). Until recently, reported use of POD data was confined to scientific studies, though various organisations are now considering their integration into operational pipelines. Wider adoption by the scientific community is currently constrained by accessibility and dissemination— only a small number of missions release near-real-time POD data for LEO satellites [55, 65]. Nevertheless, if these barriers can be overcome, POD data represent a scalable and cost-effective pathway toward the dense, low-latency observational network required for next-generation thermospheric density modelling.

### GNSS-Derived Density: A New Opportunity

Mega-constellations carry the potential to deliver the very asset assimilative frameworks need for a paradigm-shifting change: abundant, high spatial and temporal resolution, high-cadence measurements. Because a POD solution embeds the cumulative effect of drag (i.e. the integrated energy loss) experienced by a satellite along its trajectory, if it is of high enough quality, it has the potential to be inverted into a time series of orbit-averaged densities (one estimate per orbital arc, where in this work, an arc is a contiguous segment of the trajectory used in a single fit) that ought to outperform TLE-based density inversions in terms of spatial and temporal resolution. This differs from the common POD practice of co-estimating a single density scale factor per orbital arc; here, the arc-wise energy budget is inverted directly into a density estimate, so each arc yields an independent data point in the time series. A further complication is the well-known ambiguity between mass density and the satellite ballistic coefficient ($C_D A/m$): the drag signal constrains only their product, so errors in assumed $C_D$ or *A*/*m* propagate directly into the retrieved density. The fidelity of those densities, however, rests on the signal-to-noise ratio of the drag embedded in the POD data. This signal-to-noise ratio is itself dependent on several factors: the intrinsic accuracy of the POD solution (e.g. susceptible to GNSS receiver noise, processing strategy, attitude knowledge, force-mismodelling) and the strength of the drag signal. Satellites orbiting at lower altitudes or through geomagnetically disturbed conditions, where atmospheric drag presents a higher disturbing acceleration, will result in improved density retrieval quality, whereas high-altitude, quiet-time arcs tend to yield weaker, noisier estimates [63].

Development efforts towards any future models which aim to leverage this rich set of signals-of-opportunity must therefore account for these different sources of error if POD-derived observations are to realise their full potential.

### Scope of This Study

Effective use of POD data streams for thermospheric mass density estimation requires a clear understanding of the variables that govern retrieval performance. Both satellite operators and model developers must anticipate, at least approximately, the accuracy attainable from different data sources to design systems that meet operational requirements. Although accuracy and precision thresholds for orbit prediction and conjunction assessment are frequently discussed in the literature [1, 6, 16, 61], analyses of the specific factors driving density-retrieval error remain limited [63]. Establishing these relationships is essential for propagating performance requirements back to data quality, assimilation parameters, and satellite sampling strategies. This study therefore provides a quantitative foundation intended both as a blueprint for further analyses and as a set of practical rules of thumb to guide the design of next-generation density assimilation systems.

We address this objective through controlled simulations that quantify how orbit-averaged density-retrieval error, obtained via the Energy Dissipation Rate (EDR) method applied to POD trajectories, depends on four principal variables: (1) the noise characteristics of the POD products, (2) the magnitude of the drag acceleration the satellites are experiencing, (3) the number of orbital revolutions over which a single density observation is estimated (also referred to as the “fit-span"), and (4) the number of satellites contributing to each observation. We further examine how assimilation modifies these dependencies when the retrieved densities are integrated into a background model. This study uses the EDR method both directly on POD data and within an Ensemble Kalman Filter (EnKF) assimilation framework. The EDR method itself has been validated against accelerometer-derived densities and empirical models [27, 68, 70]. The combined EDR + EnKF pipeline used here, including the per-satellite $A_p$-correction strategy, has been independently benchmarked by [52], who compared retrieved densities against accelerometer data from CHAMP (∼350 km), Swarm-A/B/C (∼460–520 km), and GRACE-FO (∼490 km), as well as against MSIS and HASDM, across the May 2005 G4 storms (solar-cycle 23 declining phase, solar minimum) and the May 2024 Gannon G5 storm (solar maximum), reporting strong agreement at perigee altitudes of ∼200–750 km. The present work builds on these validated methods to characterise how density-retrieval errors scale with the controllable parameters of POD noise level, drag acceleration, fit-span, and satellite count.

To this end, we develop a more realistic representation of POD noise than the prevailing white-noise assumption, constructing three representative noise tiers (“low"/“medium"/“high") informed by values reported in the literature. These noise levels are superimposed on a set of 18 reference ephemerides propagated along Starlink-like trajectories using the HASDM density model over the month of April 2023. The resulting synthetic datasets allow us to evaluate and quantify the impacts of POD noise, drag strength, fit-span, and satellite count on retrieval performance.

Specifically, we ask:

- How do density retrieval errors respond to a more realistic POD noise model?
- How do density retrieval errors scale with fit-span ?
- How do density retrieval errors change as independent estimates from additional co-orbital satellites are averaged?
- How do these behaviours differ when densities are processed with and without an assimilative scheme?

## Experimental Setup

**Fig. 1:**
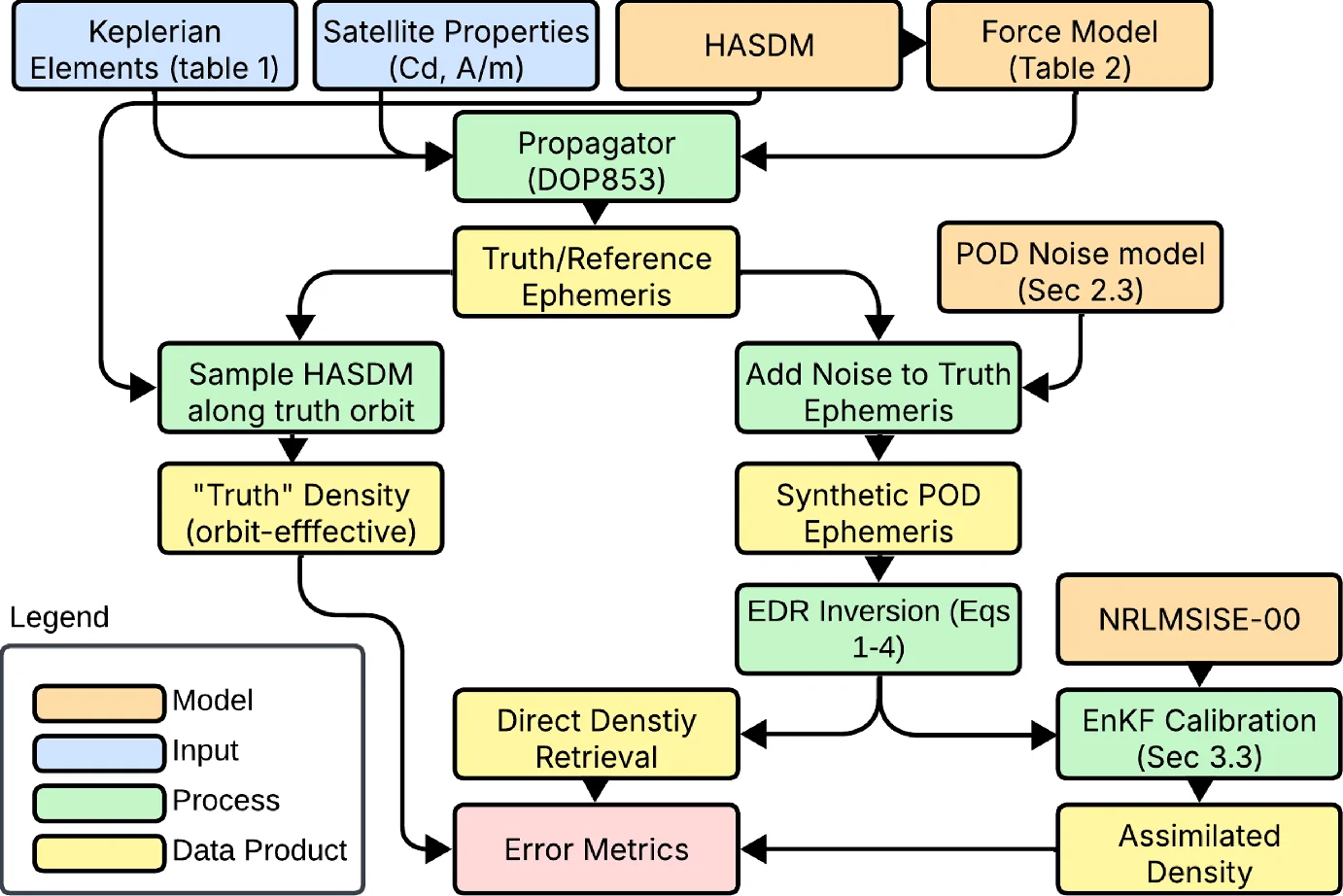
Overview of the experimental pipeline. The left branch generates the “truth” density by sampling HASDM along the propagated orbit. The right branch creates synthetic POD observations by adding a higher-fidelity noise model to the truth ephemeris, which are then inverted via the EDR method. Retrieved densities are evaluated both directly and after per-satellite Ensemble Kalman Filter (EnKF) calibration of NRLMSISE-00. Both outputs are compared against the orbit-averaged HASDM truth density

Figure 1 provides an overview of the experimental pipeline. The remainder of this section describes each stage in detail.

### Satellite Selection and Reference Data Generation

We begin by selecting one platform around which to focus: the Starlink V1.0 platform. The Starlink V1.0 satellite currently represents one of the major large-scale commercial constellations in orbit for which some information is available in the scientific literature [28]. Orbital elements for this constellation vehicle were obtained from Two-Line Elements, accessed through space-track.org. An effective area-to-mass ratio of $A/m=0.05\,\hbox {m}^2\,\hbox {kg}^{-1}$ was adopted from [62] and used throughout our simulations (Table 2). A constant $C_D=2.2$ is assumed; because $C_D$ is defined with respect to the same reference area used in the drag computation, uncertainties in $C_D$ map nearly one-to-one onto retrieved density errors [62].

“Truth" ephemerides are then generated by propagating the Keplerian elements in Table  1 using the numerical integrator and force model summarized in Table 2. Unless otherwise specified, all analyses and results pertain to 1–7 April 2023. The $F_{10.7}$ index at this time ranged from 125.1 solar flux units (sfu) to 137.6 sfu representing a medium solar activity period. The first week (1–7 April) is geomagnetically quiet, with a peak Kp index of $K_p=4^{-}$. The second week (21–28 April) encompasses a G4-class geomagnetic storm that peaked at $K_p=8^{+}$ on 23 April, followed by a multi-day recovery phase. Week-long analysis windows were selected for two reasons: first, to maintain computational tractability when sweeping across the full parameter space of noise levels, drag-acceleration levels, fit-spans, and satellite counts; and second, to allow targeted sampling of both geomagnetically quiet and severe storm-time conditions within the same month. More extensive comparisons of the EDR retrieval method against HASDM and MSIS over longer time periods are provided in [52].

Section 3.3 investigates the simultaneous use of multiple satellites spread along a common orbital track to resolve densities. Ephemerides for these additional satellites were generated synthetically by replicating the reference orbit and offsetting only the true anomaly. In practice, this means that each satellite follows an identical trajectory, shifted in phase along the orbit so that the group forms a uniformly spaced “string of pearls” configuration. For N=18, for example, the satellites are spaced $20^{\circ }$ apart in true anomaly, ensuring uniform coverage of the orbit. The aim of this setup is to isolate the effect of satellite count on density retrievals by holding all other orbital parameters constant.

Geometric properties ($C_D$ and *A*/*m*) are held constant throughout each simulation. Although a time-varying attitude model would increase physical fidelity [27, 28], it was omitted to focus on the relationship between density retrieval errors and POD model errors.

**Table 1:** Initial Keplerian elements of the simulated Starlink-like satellite

Satellite	*a* (km)	*h* (km)	*e*	*i* ()	Ω ()	ω ()	*M* ()
Starlink V1.0	6928.1	∼550	0.00001	53.0	125.0	90.0	40.0

*a*: semi-major axis; *h*: approximate altitude above the WGS-84 ellipsoid; *e*: eccentricity; *i*: inclination; Ω: right ascension of the ascending node; ω: argument of perigee; *M*: mean anomaly

**Table 2:** Force model and numerical integrator

Component	Description	Reference
Numerical Integrator	Dormand–Prince (DOP853), variable step size	[21]
Earth Gravity	EGM2008 to degree and order 120	[58]
Third-Body Perturbations	Sun and Moon point-mass gravity	[29]
Solar Radiation Pressure (SRP)	Cannonball model, Cone shadow model	[45]
Atmospheric Density Model	HASDM	[68]
Geometric Model	0.05 (for both SRP and Drag), Panel Model	[27, 62]

Earth radiation pressure and thermal re-radiation were not included in the force model. Because the simulation period falls near solar maximum, drag is the dominant non-conservative force and these radiative perturbations are comparatively small. At lower points in the solar cycle, however, drag weakens while radiation pressure remains largely unchanged, and these contributions become much more significant relative to drag [62, 73]. Incorporating Earth radiation pressure and thermal re-radiation into the force model would improve physical fidelity, particularly for solar-minimum conditions, and is left to future work.

The reference (or “truth") density time series against which benchmarking of the different strategies is carried out was generated by sampling the HASDM densities along each propagated orbit and computing the mean density over the period corresponding to one full orbital revolution of the satellite.

### Energy Dissipation Rate Based Density Retrieval

Whilst a number of approaches exist for estimating density from POD data, we focus on the EDR method. The application of this method to GNSS-enabled small satellites for thermospheric density retrieval was demonstrated by [70]. The EDR method can be applied to many different kinds of observations and is computationally more tractable than alternative methods such as POD accelerometry [63]. The use of this kind of method underpins state-of-the-art models such the initial version of HASDM [68] and has been applied to POD data across the scientific literature [27, 53, 54, 69] providing valuable sources of comparison.

We follow the method outlined below:

For an Earth-centred, Earth-fixed (ECEF) position $\textbf{r}=(x,y,z)$ and velocity $\textbf{v}=(\dot{x},\dot{y},\dot{z})$, the orbit energy is

1 $$\begin{aligned} E(t) = \frac{{\nu ^{2} }}{2} - \frac{{\omega _{ \oplus }^{2} }}{2}{\hspace{0.55542pt}} (x^{2} + y^{2} ) - \frac{\mu }{r} - U_{{{\text {nsph}}}} ({\textbf{r}}) \end{aligned}$$

with $r=\Vert \textbf{r}\Vert$, $\nu =\Vert \textbf{v}\Vert$, $\mu =3.986004418\times 10^{14}\,\mathrm {m^{3}\,s^{-2}}$ is the Earth’s standard gravitational parameter, and $\omega _\oplus =7.2921159\times 10^{-5}\,\mathrm {rad\,s^{-1}}$ is the Earth’s angular velocity. The non-spherical potential $U_{\text {nsph}}$ is evaluated from the EGM2008 gravity field (evaluated to degree and order 120).

Assuming the atmosphere co-rotates with Earth, the relative-wind (free-stream) velocity is given by

$$ \textbf{v}_{\text {rel}}=\textbf{v}-\boldsymbol{\omega }_\oplus \times \textbf{r}\qquad v_{\text {rel}}=\Vert \mathbf \nu _{{{\text {rel}}}}\Vert $$

The drag force on the satellite is

$$ F_{\text {drag}}=\tfrac{1}{2}\,C_D\,A_\text {ref}(t)\,\rho (t)\,v_{\text {rel}}^{2} $$

and the corresponding drag acceleration is $a_{\text {drag}}=F_{\text {drag}}/m$. Under the assumption that drag is the dominant source of dissipation of orbital energy over one complete orbit, the power dissipated is $F_{\text {drag}}\,v_{\text {rel}}$, giving the rate of energy dissipation [70]

2 $$\begin{aligned} -\frac{\mathrm dE}{\mathrm dt}= \frac{1}{2m}\,C_D\,A_\text {ref}(t)\, \rho (t)\,v_{\text {rel}}^{3} \end{aligned}$$

where m is spacecraft mass, $C_D$ the drag coefficient, and $A_\text {ref}(t)$ the ram profile of the spacecraft in the direction of the oncoming atmospheric flux: $\textbf{v}_{\text {rel}}$.

Integrating between successive perigees from $t_0$ to $t_1$,

3 $$\begin{aligned} \Delta E =E(t_1)-E(t_0) =-\frac{\rho _\text {eff}}{2m} \int _{t_0}^{t_1} C_D A_\text {ref}\,v_{\text {rel}}^{3}\,\mathrm dt \end{aligned}$$

Rearranging for the orbit-effective density $\rho _\text {eff}$, which by definition is the constant that, when placed outside the integral, reproduces the observed energy change:

4 $$\begin{aligned} \rho _\text {eff}=-\frac{2m\,\Delta E}{\displaystyle \ \int _{t_0}^{t_1}C_D A_\text {ref}\,v_{\text {rel}}^{3}\,\mathrm dt} \end{aligned}$$

Perigees are detected by locating local minima in the radial distance r(t) where $\dot{r}$ changes sign from negative to positive. To suppress false triggers in noisy POD data, a candidate is accepted only if r subsequently increases by at least $2\;\text {m}$ over a minute-long window.

### POD Noise Model

POD solution noise is one of the primary mechanisms through which the quality of density retrievals are degraded. In previous studies looking at modelling the effect of POD noise on density retrieval, POD noise errors were assumed to be isotropic white noise [62, 63]. We aim to refine that assumption to provide a noise model which is more representative of the reported decimetre- to metre-level performance typical of commercial small-sat platforms [4]. Unfortunately, the open literature on commercial GNSS-based POD solutions is sparse, arguably a key reason the white-noise modelling approach persists in the literature. [66] present an excellent meta-analysis of science-grade LEO-POD performance, yet no comparable survey exists for the commercial sector. To the best of the authors’ knowledge, to date, detailed metrics of a commercial constellation’s POD product exist in the public domain only for the Spire satellites [4]; we therefore combine those figures with the Sentinel “navigation” solution [36] as a plausible lower bound on what a well-resourced commercial operator might achieve, while recognising that many fleets are likely to deliver lower accuracy.

Sentinel-1/-2/-6 near-real-time (NRT) orbits, validated via International Laser Ranging Service (ILRS) data, exhibit ∼0.3–$0.7\;\text {m}$ three-dimensional (3-D) root-mean-square (RMS) position error, whereas their science-grade orbits remain below $7\;\text {cm}$ [36, 48]. Spire’s commercial POD product lies in between 27–$30\;\text {cm}$ 3D RMS when compared against Bernese/NAPEOS references that agree at the 6–$7\;\text {cm}$ level [4]. For comparison with commercial uncooperative tracking data, at the time of writing (August 2025), LeoLabs list $41\;\text {m}$ accuracy and $16\;\text {m}$ precision for their uncooperatively derived data (as reported at https://platform.leolabs.space/system_metrics).

Across the reviewed literature characterizing LEO POD solutions [4, 32, 36, 46–48], along-track errors consistently dominate and tend to follow a 1/f (“pink-noise”) spectrum; cross-track errors are then smaller and more isotropic, and radial errors tend to be the smallest and are also largely isotropic [36]. The errors in POD solutions display temporal autocorrelation. In the case of Sentinel-1 and -2’s “nav" solutions this behaviour can be captured by a first-order autoregressive process (AR(1)) with 600–$800\;\text {s}$ decay. [36] also cite that the variance is heteroscedastic, oscillating in tandem with geometric dilution of precision (GDOP) at 0.5–$3\;\text {m}$ peak-to-peak. Along-track bias is also commonly reported (e.g. -0.2 to $-0.5\;\text {m}$ for Sentinel-2A and B [36]).

The sources of these POD errors are numerous and may be attributed to effects such as: receiver noise and multipath [48, 66], ionospheric and/or tropospheric delay, and mismodelled accelerations [3].

Note that most science missions benefit from being able to routinely verify their POD solutions against a mm-level accuracy, independent measurement source. For example, SLR. However, the presence of retro-reflectors is exceedingly rare on commercial satellites thus requiring different and less robust validation approaches (e.g. orbit overlaps). For instance [4] validate POD solutions from the Spire constellation against two independent POD processing software packages (Bernese, NAPEOS). This provides a relative rather than fully absolute verification. The absence of SLR or equivalent truth data complicates both error characterisation and performance guarantees for commercial fleets.

**Fig. 2:**
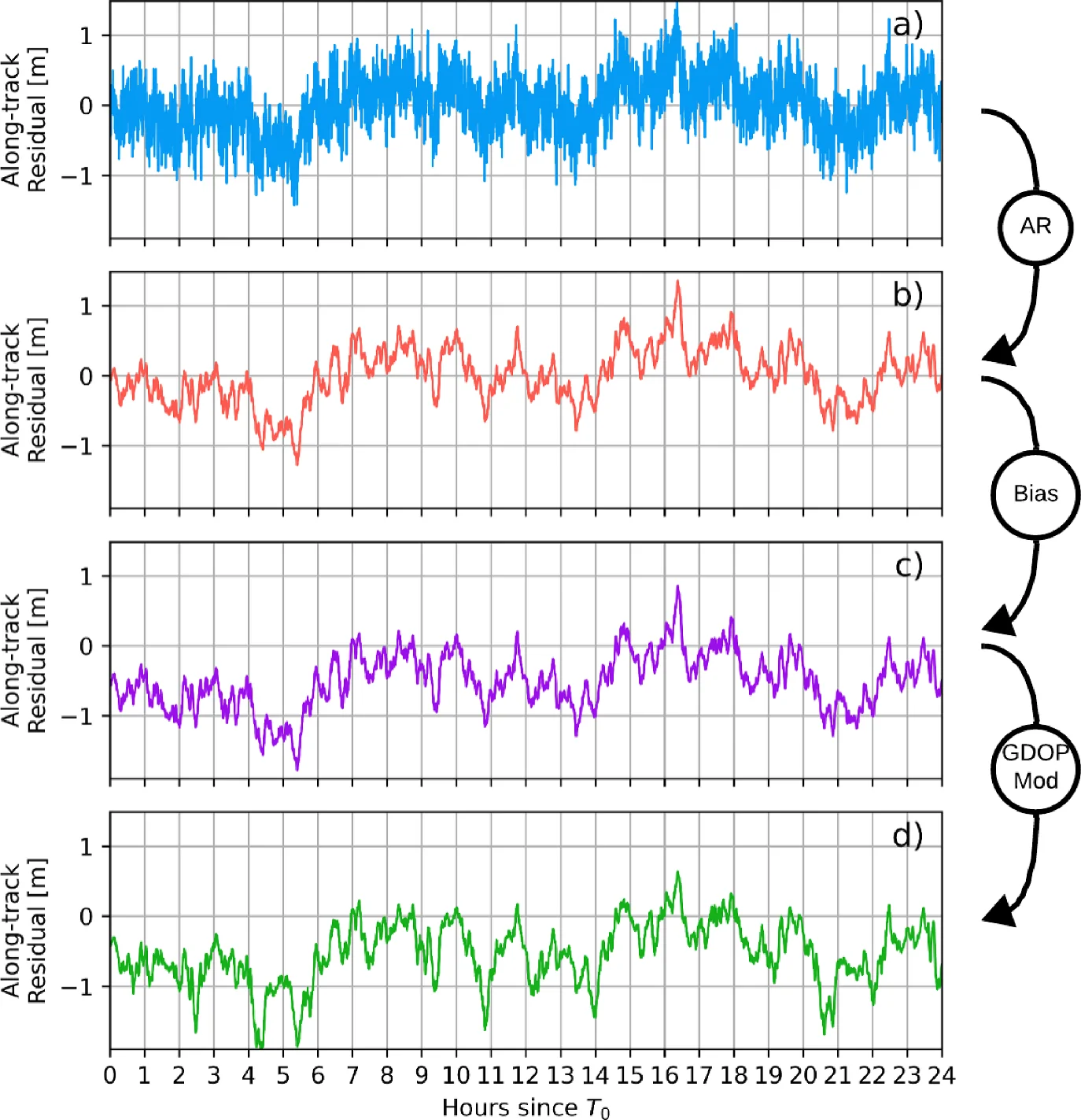
Along-track residuals of a 24-hour trajectory arc with respect to the reference orbit (Sect. 2.1). Going from top to bottom, each graph corresponds to the addition of a new component of noise. Panel **a** shows the modulation of the white noise into pink noise. Panel **b** shows the effect of the application of the autocorrelation model (AR(1)). Panel **c** shows the effect of adding a −0.5 m bias. Panel **d** shows the effect of GDOP-modulation of the signal

To reproduce the dominant signatures reported in the LEO POD noise characterization literature, a synthetic noise model is overlaid onto each state vector within the reference ephemerides (Sect.2.1). The noise model is composed of the following components: anisotropic noise, pink (1/*f*) colouring in the along-track direction, short-range autocorrelation, bias, and heteroscedastic scaling of the noise as a function of GDOP [36, 49]. Figure 2 illustrates the effect of each step on the along-track residuals of a 24-hour arc of simulated POD data when compared to the “truth" simulated reference ephemeris We outline the method to generate and apply this noise model below:

For each epoch t we draw a zero-mean position residual

$$ \boldsymbol{\varepsilon }_w(t)= \begin{bmatrix}\varepsilon _H\\ \varepsilon _C\\ \varepsilon _L\end{bmatrix} \sim \mathscr {N}\!\bigl (\textbf{0},\, \operatorname {diag}(\sigma _H^2,\sigma _C^2,\sigma _L^2)\bigr ) $$

where the standard deviations $\sigma _{H,C,L}$ (height, cross-track, along-track) of each noise model tier (high, medium, low) are defined based on values in the literature (values presented in Table 3).

The along-track component is coloured by a frequency-domain filter (meaning the noise is adjusted so that slow oscillations become stronger and rapid oscillations weaker)

$$ \widehat{\varepsilon }_{\text {pink}}(f)\;=\; \widehat{\varepsilon }_w(f)\; \bigl (f/f_0\bigr )^{-1/2} $$

where hats denote Fourier coefficients and $f_0$ is the fundamental frequency of the window. An inverse Fast Fourier Transform returns the time-domain series $\varepsilon _{\text {pink}}(t)$, which replaces $\varepsilon _L$.

Short-range correlation is imposed with a first-order autoregressive process,

$$ x_t = \alpha \,x_{t-1}+e_t\qquad e_t\sim \mathscr {N}(0,\sigma ^2) $$

using a 10-s step size and a target folding time of $k_{\text {decay}}=70$ samples ($=700\;\text {s}$), as noted in [36].

$$ \alpha \;=\; 1-\frac{1}{k_{\text {decay}}}\;=\;0.9857 $$

The AR(1) operator is applied to each positional axis (H/C/L).

A constant offset $b_L=-0.5\;\text {m}$ is added in the along-track of all solutions, aligning with the mean bias reported for Sentinel NRT solutions [36].

As per [36], POD errors tend to be modulated by GDOP, which is caused by variations in the geometry of the overhead GNSS satellites that the LEO orbiters use to generate their own POD solutions. We simulate reception from a dual GPS/Galileo constellation by propagating broadcast ephemerides [23, 64] over the period of interest. To ensure robust visibility and geometry, GNSS satellites with elevation angles above $5^\circ$ are retained, following standard practice in space-borne GNSS-POD processing [14]. The receiver antenna is assumed to be zenith-facing and nadir-mounted, a common configuration on commercial LEO spacecraft [4].

With line-of-sight unit vectors $\textbf{u}_i(t)$ to n visible satellites, the design matrix is $A=[\textbf{u}_1^{\!\top }\;\,\dots \;\,\textbf{u}_n^{\!\top }]^{\!\top }$ and the GDOP is

$$ g(t)\;=\;\sqrt{\operatorname {tr}\!\bigl [(A^{\!\top }A)^{-1}\bigr ]} $$

Let $\tilde{g}=\operatorname {median}\{g(t)\}$. We select an exponential heteroscedastic factor

$$ m(t)\;=\;\exp \!\bigl [g(t)-\tilde{g}\bigr ] $$

consistent with the multiplicative behaviour of GDOP observed by [36]. The final position error is

$$ \boldsymbol{\eta }(t)\;=\;m(t)\, \bigl [\boldsymbol{\varepsilon }_w(t)_{\text {(coloured)}}\bigr ] +\begin{bmatrix}0\\ 0\\ b_L\end{bmatrix} $$

For each simulated ephemeris in this study, three different POD noise solution tiers are created. The magnitude of the noise associated with these three noise tiers were selected in an attempt to reflect the range of solutions found in the literature and those that may be encountered by commercial POD data providers. The values selected are listed in Table 3.

**Table 3:** H/C/L error used for each noise model, corresponding 3D RMS error, and source for selected values

Noise model	Height	Cross track	Along track	3D RMS	Reference/Justification
Low	0.2	0.1	0.2	0.300	[36]
Medium	0.1	0.2	0.4	0.458	[4]
High	0.8	0.4	1.6	1.830	∼4× Medium case

### Comparison to Real POD Residuals

Whilst the noise modelling strategy (Sect. 2.3) has been developed based on characteristics reported in the literature [36], we now aim to further assess whether the proposed noise model produces realistic POD error characteristics. To do so, we compare it against time series of residuals between “navigation” and “precise” orbit solutions from Swarm-A, following the approach outlined in [36]. We compute residuals by differencing the onboard navigation solution (SW_GPSA_NAV_1B) from the precise science orbit (SW_MODA_SC_1B) over an 8.5-hour interval on 5 August 2025.

The resulting 1-σ errors in the radial/cross-track/along-track frame were 1.179 m, 0.450 m, and 0.565 m respectively, yielding a 3D RMS of 1.38 m. The radial component dominates the error budget, which is atypical for LEO POD residuals where along-track errors generally dominate [66]. This may be attributable to Swarm’s low area-to-mass ratio, which was designed to minimise drag perturbations [33], or to the characteristics of the onboard navigation filter.

**Fig. 3:**
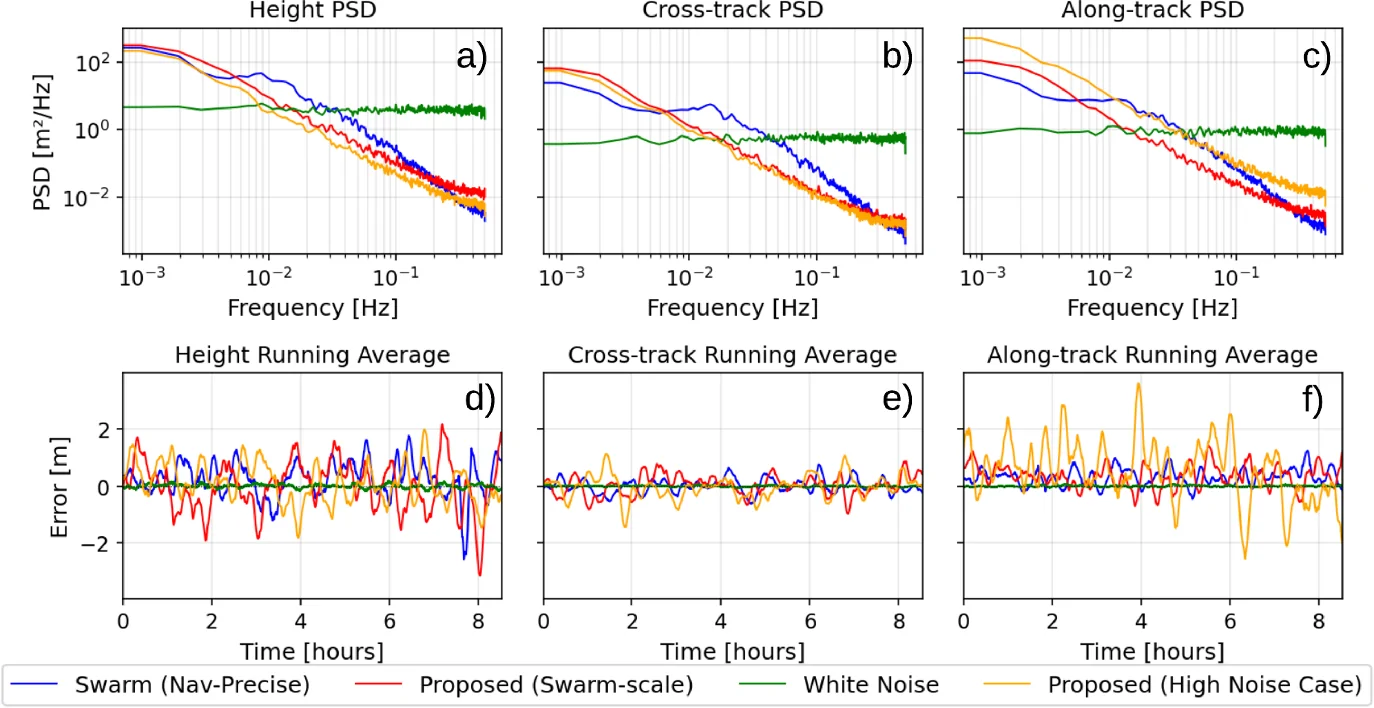
Comparison of Swarm-A navigation–precise residuals (blue) with the proposed noise model scaled to match Swarm 1-σ errors (red), white noise scaled to match the 3D RMS (green), and the proposed noise model in the high-noise configuration (orange). All residuals are computed relative to the Swarm-A precise orbit (SW_MODA_SC_1B) over an 8.5-hour interval on 5 August 2025. Top row **a–c**: power spectral density. Bottom row **d–f**: 10-minute running average. Left, centre, and right columns correspond to radial, cross-track, and along-track components respectively

Figure 3 compares the Swarm residuals against synthetic noise time series generated using the procedure described in Sect. 2.3 and overlaid onto the Swarm precise orbit. Four cases are shown: (1) the Swarm navigation–precise residuals (blue), (2) the proposed noise model, scaled component-wise to match the observed Swarm 1-σ errors in each axis (red), (3) simple white noise, scaled isotropically to match the observed 3D RMS (green), and (4) the proposed noise model in the high-noise configuration (orange), where the along-track error dominates as is more typical for LEO POD solutions.

The power spectral densities (panels a–c) illustrate that the proposed noise model better approximates the 1/*f* spectral slope characteristic of real POD errors, whereas white noise exhibits a flat spectrum. Discrepancies remain in the $10^{-2}$–$10^{-1}$ Hz band (periods of 10–100 s) between the proposed model and the Swarm data. These may reflect effects not captured in our model such as multipath, receiver tracking dynamics, attitude variations, or ionospheric scintillation.

Panels d–f display the time series of errors as 10-minute running averages. We select this presentation rather than the native 1 Hz time series for two reasons: firstly to improve legibility, and secondly for comparability with Figures 3 and 4 of [36], which use the same rolling average method. Qualitatively, these panels show that the proposed model exhibits similar low-frequency structure to the Swarm residuals, while the white noise averages to near-zero throughout. Exact phase alignment between the synthetic and observed signals is not expected, since the noise generation process is partly stochastic (though the GDOP modulation component is deterministic).

The proposed noise model reproduces the spectral characteristics and temporal structure of real POD residuals more faithfully than white noise. A more quantitative validation, and one more representative of commercial constellation operations, would require access to commercial-grade POD data with known processing characteristics. To the authors’ knowledge, no such dataset is publicly available. We also note that the Swarm receiver is a scientific-grade instrument, and as such of higher quality than typical commercial hardware. It is therefore possible that it may outperform commercial-grade receivers even under similar processing strategies. Nevertheless, this comparison supports the conclusion that the proposed noise modelling approach represents a step in the direction of higher physical fidelity over the white-noise assumption commonly adopted in the literature.

### Effect of Drag Acceleration on Retrieval Error

**Fig. 4:**
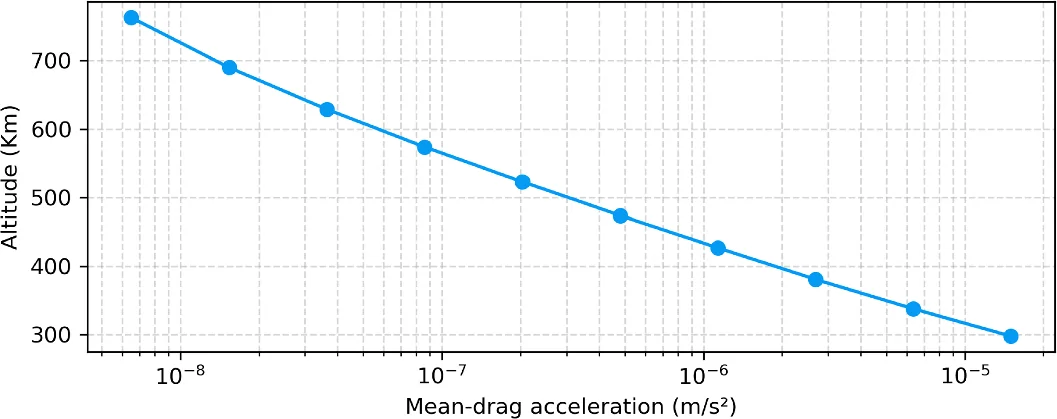
Mean altitude and corresponding drag-acceleration of each of the 10 altitudes sampled over 1–7 April 2023. Densities sampled from the HASDM density model

Ten drag-acceleration levels were sampled. These were selected to encompass the typical range of drag-acceleration levels encountered by LEO satellite constellations across a range of solar and geomagnetic activity levels. The drag-acceleration bounds were defined as ranging from $6.5\times 10^{-8}$ to $1.5\times 10^{-5}\,\mathrm {m\,s^{-2}}$. Given the broadly exponential relationship between thermospheric density and altitude [45], the samples were spaced uniformly in logarithmic space. Since $C_D$ and *A*/*m* were kept constant, variation in the semimajor-axis was the only variable modified to sample the drag-acceleration space. Figure 4 shows the sampled altitudes and corresponding mean orbital drag accelerations over 1–7 April 2023 for each sample.

### Effect of Fit-Span on Retrieval Error

The fit-span parameter used in the EDR method effectively represents the amount of time over which the integral is carried out (Eq.3). Increasing the fit-span reduces the noise by increasing the length of the observation and thus the signal of any given density estimate. This process will also result in a smoothing of the extracted density signal as the result of the integral is now attributed to a longer orbital arc. In the results presented, we varied this over integer values from 1 to 5 orbits. For each fit-span N, the change in orbital energy due to drag was measured by integrating over N consecutive orbits using the simulated trajectories over which the different POD noise levels were overlaid. The retrieved density time series for each span was then compared to the reference density data to measure the RMS% error. Note that when studying the impact of increasing fit-span we compared the mean HASDM density over the same N-orbit interval. This procedure ensures that comparisons across fit spans are performed on a consistent temporal averaging basis, however, this means that the loss of temporal resolution which occurs as a result of increasing the fit-span is not accounted for.

### Assimilating POD-Derived Densities

POD data alone enable the provision of density estimates along the orbits of the satellites for which the POD data is available. However, most applications that satellite operators and Space Situational Awareness (SSA) service providers are interested in (e.g. collision avoidance, orbit propagation) typically require the use of density values spanning much larger spatial scales (potentially across the entirety of LEO). Leveraging observations to this end is typically achieved through an assimilation scheme which makes use of the observations- in this case POD-derived EDRs- to calibrate a background model. In principle, density scale factors co-estimated within the POD process itself could also be applied to correct the background model globally; here, however, we treat the POD solution as fixed and perform the calibration as a separate post-processing step. Because NRLMSISE-00 is an empirical model that does not propagate atmospheric state forward in time, the scheme presented here estimates corrections to model inputs (specifically, the $A_p$ geomagnetic index) rather than to atmospheric state variables. This approach is more accurately described as model calibration than state estimation in the data assimilation sense, and its results should not be directly generalised to state-estimation frameworks applied to General Circulation Models, where the estimated state influences future predictions. The following section is provided under the expectation that future density models will rely heavily on POD-derived densities within such frameworks.

The process of assimilating density observations into a background model will warp the relationship between retrieval error and the underlying variables of fit-span, drag-strength, POD quality, and satellite count. This section aims to provide an assessment of the impact of assimilation on the density retrieval errors.

During geomagnetically quiet periods and over relatively short time spans (≤ a few days), orbit errors can often be addressed through static bias adjustments (e.g. ballistic coefficient estimation), such that the assimilation process may become akin to a bias tuning exercise. In contrast, storm-time conditions introduce steep temporal gradients in density. These features stress-test the ability of assimilation schemes to resolve time-dependent corrections rather than simply absorb static biases, making the comparison more discriminative and operationally relevant. For this reason, we focus this section of the analysis on the week spanning 21st–28th April 2023. This week-long window includes a geomagnetically quiet phase and a G4-class geomagnetic storm that peaked at $K_p=8^{+}$ on 23 April (including main phase and post-storm recovery).

The assimilation process used in this section of the work is outlined below. It follows the implementation in [52], where the same per-satellite $A_p$-correction strategy was benchmarked against accelerometer-derived densities from CHAMP, Swarm, and GRACE-FO across the May 2005 G4 storms and the May 2024 Gannon G5 storm, demonstrating that the correction recovers storm-time density variability at perigee altitudes of ∼200–750 km. Orbit-averaged EDRs are assimilated into NRLMSISE-00 using the Ensemble Kalman Filter (EnKF). The filter estimates a correction series $\Delta A_p(t)$ that perturbs the 3-hourly geomagnetic index $A_p$; all other inputs remain fixed, apart from an empirically selected solar–flux correction. A value of $\Delta F_{10.7}=-40\;\text {sfu}$ was empirically selected to absorb the baseline density bias in NRLMSISE-00 over the simulation period (April 2023), allowing the EnKF to focus on estimating shorter-timescale geomagnetic corrections via $\Delta A_p$.

A 48-hour window is sampled every $\Delta t_\text {step}=3$ h. At epoch $t_k$, the state vector collects the present and next three $A_p$-corrections,

$$ \textbf{x}_k=\bigl [\Delta A_p(t_k),\;\Delta A_p(t_{k+1}),\;\Delta A_p(t_{k+2}),\;\Delta A_p(t_{k+3})\bigr ]^{\!\top }\in \mathbb {R}^{4} $$

so the sliding estimation window influencing any analysis spans 12 h. Although the filter formalism treats $\Delta A_p$ as a state vector, this quantity is an empirical model input parameter, not a physical atmospheric state variable. The distinction is important: because NRLMSISE-00 does not propagate atmospheric state forward in time, the estimated corrections do not influence future model predictions in the way that state variables would in a general circulation model. The estimated $\Delta A_p$ correction should be interpreted as an effective parameter adjustment that reduces NRLMSISE-00 density errors along the satellite orbit, not as a correction to the actual observed geomagnetic index. This adjusted $A_p$ should not be used to drive other density models. The sliding window moves forward by 3 h after each estimation epoch. Because consecutive states overlap by three elements, every epoch is re-analysed as new data arrive and no temporal gaps form. Between analyses the correction field is assumed constant, $\textbf{x}_{k+1}^{f}=\textbf{x}_{k}^{a}$. To maintain ensemble spread, the newest element is redrawn at each step from $\mathscr {N}(0,\sigma _{A_p}^{2})$ with $\sigma _{A_p}=50$. The initial state is $\Delta A_p=0$ (i.e. no correction). The value $\sigma _{A_p}=50$ was chosen to be large enough to allow the filter to track the full range of $A_p$ variations encountered during storm conditions. The ensemble size is $N_e=36$.

Each EDR measurement represents the density averaged over a complete orbital arc centred on time $t_j$. For ensemble member i, the corresponding model prediction is obtained by integrating density returned by NRLMSISE-00 along that arc while applying $\Delta A_p$ and $\Delta F_{10.7}$,

$$ H_j(\textbf{x}_i)=\overline{\rho }_{\text {NRLMSISE-00}}\!\Bigl (t_j;\;A_p(t_j)+\Delta A_p(t_j),\;F_{10.7}+\Delta F_{10.7}\Bigr ) $$

**Fig. 5:**
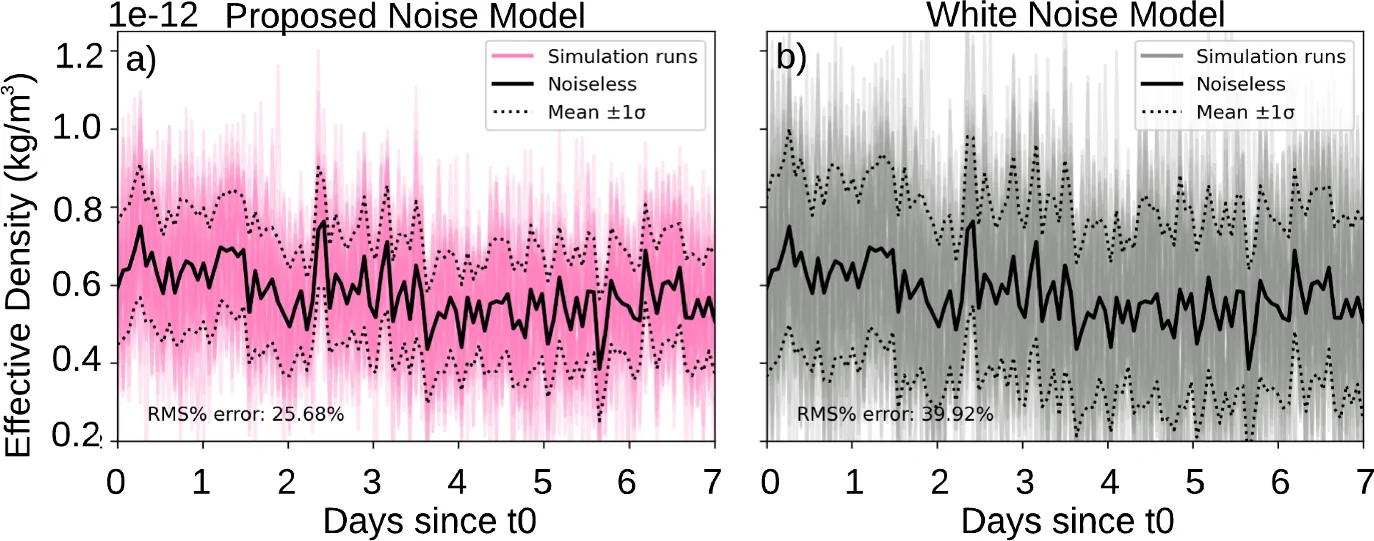
Monte-Carlo Sampling of the error associated with different noise modelling approaches across the Starlink-like reference trajectory over 1–7 April 2023. Panel **a** shows the error for the higher-fidelity noise model outlined in Sect.2.3. Panel **b** shows the error for a white noise model. Both models are equivalent in 3D RMS terms (0.458 m). Each panel contains 50 samples of each noise model

Let $\textbf{y}\in \mathbb {R}^{m}$ stack the $m=6\text {-}7$ orbit-mean densities that fall within the current window, and let $\textbf{H}(\textbf{x}_i)$ be the corresponding model outputs for ensemble member i. Although the experiment is simulated and the uncertainties can be accurately characterised, we deliberately emulate the operational setting where per-arc uncertainties are not known accurately a priori: we therefore fix a single relative observation uncertainty for all arcs, chosen as an intermediate value between the two representative levels shown in Fig. 5. We adopt a one–sigma fractional error of 30% for every orbit-mean density, yielding the observation error covariance matrix

$$ \textbf{R}\;=\; \textrm{diag}\!\bigl ((\alpha \,y_1)^2,\ldots ,(\alpha \,y_m)^2\bigr )\qquad \alpha =0.30 $$

We use normalised ensemble perturbations $\textbf{X}',\textbf{Y}'$ (columns scaled by $1/\sqrt{N_e-1}$) so that the sample covariances are $\textbf{P}_{xy}=\textbf{X}'\textbf{Y}'^{\!\top }$ and $\textbf{P}_{yy}=\textbf{Y}'\textbf{Y}'^{\!\top }$. The EnKF analysis is given by [5, 25, 38, 72]:

$$ \textbf{K}\;=\; \textbf{P}_{xy}\,\bigl (\textbf{P}_{yy}+\textbf{R}\bigr )^{-1}, \qquad \textbf{x}_i^{a} \;=\; \textbf{x}_i^{f} + \textbf{K}\bigl (\textbf{y}-\textbf{H}(\textbf{x}_i^{f})\bigr ) $$

This implementation uses the stochastic EnKF, in which each ensemble member’s observation vector is perturbed by a draw from the observation error distribution before computing the innovation. Deterministic alternatives such as ensemble square root filters avoid this sampling noise but were not explored here; the stochastic variant was adopted for simplicity and consistency with the implementation in [52]. Given the low state dimension (n=4) relative to the ensemble size ($N_e=36$), the sampling noise introduced by observation perturbation is negligible.

After all windows are processed, an epoch may appear in up to four analyses; their arithmetic mean yields the final estimate $\widehat{\Delta A_p}(t_k)$. At the temporal edges, where fewer analyses contribute, a linear weight proportional to order of appearance compensates for reduced redundancy without bias. The resulting $A_p$ correction time series is fed back into NRLMSISE-00 to generate the assimilated density time series.

All assimilated EDR time series generated were processed through this framework. Note that assimilation is performed on a per-satellite basis rather than jointly across all satellites; each satellite therefore produces its own independent $\Delta A_p(t)$ correction series. While extending the observation vector to assimilate multiple satellites jointly is straightforward in principle, the per-satellite approach was retained here to isolate the contribution of each spacecraft and to remain consistent with the single-satellite framework of [52]. A joint multi-satellite assimilation is left to future work.

### Aggregating Simultaneous Density Observations From Multiple Satellites

Most commercial satellite constellations tend to follow clearly defined orbital configurations (e.g. Walker-Delta [74], Flower [51]) to carry out their mission. These orbital configurations usually involve geometrically near-identical satellites orbiting along a nominal orbital path. This mode of operation opens up a further avenue of exploration for POD derived densities. At present, within existing studies, the main lever that is actioned to reduce noise and increase accuracy is to extend the measurement window (typically by increasing the fit-span). Constellation data offers a different mechanism- using a greater number of simultaneous measurements along the same orbital path. To investigate the effect of using data from more satellites on retrieving a density time series, we use the string-of-pearls configurations described in Sect. 2.1 with $N=\{1,2,3,6,9,18\}$ co-orbital satellites. With the fit-span fixed at one orbit, the EDR method is modified to provide neutral densities at predetermined epochs tied to the orbital period of the first satellite (rather than perigee-to-perigee as outlined in Sect. 2.2). This modification is required so that samples can be averaged at a common cadence. The mean retrieved density across the N satellites was then compared to the “truth" background density model: HASDM. For this section of the analysis, we use the same storm time period outlined in Sect.2.7, and compare the effects of averaging density observations from multiple satellites both with and without use of the assimilation tool to investigate the effect of noise on the density retrieval exercise.

## Results

### Impact of Improved Noise Model

Existing studies on the effect of POD noise on density retrieval performance tend to assume POD noise to be white [62, 63]. Thus, as a first point of comparison we consider the impact of modifying this commonly used approach to a higher-fidelity one (outlined in Sect. 2.3). To do so, we apply a white noise model and the noise model outlined in Sect. 2.3 to the reference ephemeris for the nominal-altitude Starlink-like satellite over 1–7 April 2023. We then use both ephemerides to generate a time series of effective densities using the EDR method (see Sect. 2.2) to analyse the effect of changing the noise model on the end product we are interested in: density time series. To ensure fair comparison, the white and higher-fidelity noise models are made equivalent in 3D RMS terms. For this experiment, the absolute noise level of both noise models is set to 0.458 m (3D RMS). For the higher-fidelity noise model, the 1-sigma anisotropic noise distribution is 0.2 m in the height direction, 0.1 m in the cross-track direction and 0.4 m in the along-track direction. For the white noise case this translates to a 1-sigma error of 0.265 m in all three directions. To generate an estimate of the RMS percentage error and uncertainty of the density time series retrieved using each noise model, we sample each noise distribution by repeating this process 50 times, each time sampling at random from both the noise distributions. Figure 5 shows the 50 density time series generated for each noise model and the density time series returned using a noise-free ephemeris.

The RMS% error is calculated as follows: Let *M* be the number of Monte-Carlo simulations (50 in this case) and *N* the number of observations in the density time series (110 in this case). We denote by

$$ \rho _{i,j},\quad i=1,\dots ,M,\;j=1,\dots ,N $$

the effective-density from the *i*th simulation at time index *j*, and by

$$ \rho _{0,j},\quad j=1,\dots ,N $$

the “noiseless” effective-density at time index *j*. Then the RMS-percentage error is computed as

5 $$\begin{aligned} \text {RMS}\% \text { error} \;=\; \frac{ \displaystyle \sqrt{ \frac{1}{MN} \sum _{i=1}^{M}\sum _{j=1}^{N} \bigl (\rho _{i,j} - \rho _{0,j}\bigr )^{2} } }{ \displaystyle \frac{1}{N}\sum _{j=1}^{N}\rho _{0,j} } \times 100\% \end{aligned}$$

We see that with both noise models being attributed identical 3D RMS noise values, the white noise model results in larger error. We attribute this in part to the decreased level of error in the height direction of the higher-fidelity noise model when compared to the white noise model. Moreover the autocorrelation present within the higher-fidelity solution likely has the effect of reducing the variance in the solution, resulting in a smaller spread around the mean.

### Effect of Drag Strength and Fit-Span

When retrieving densities from POD data along the path of a single satellite, the three key variables driving the performance of the EDR method are: POD solution quality (noise/bias), strength of the drag signal (drag acceleration), and number of orbits over which the EDR integral (Eq. 3) is carried out (fit-span). Increases in all three of these variables are expected to result in lower density retrieval error, although for fit-span this comes at the cost of reduced temporal resolution. In the following section we investigate whether this result holds and to what extent. From this point on, all noise is modelled using the higher-fidelity noise model outlined in Sect. 2.3.

**Fig. 6:**
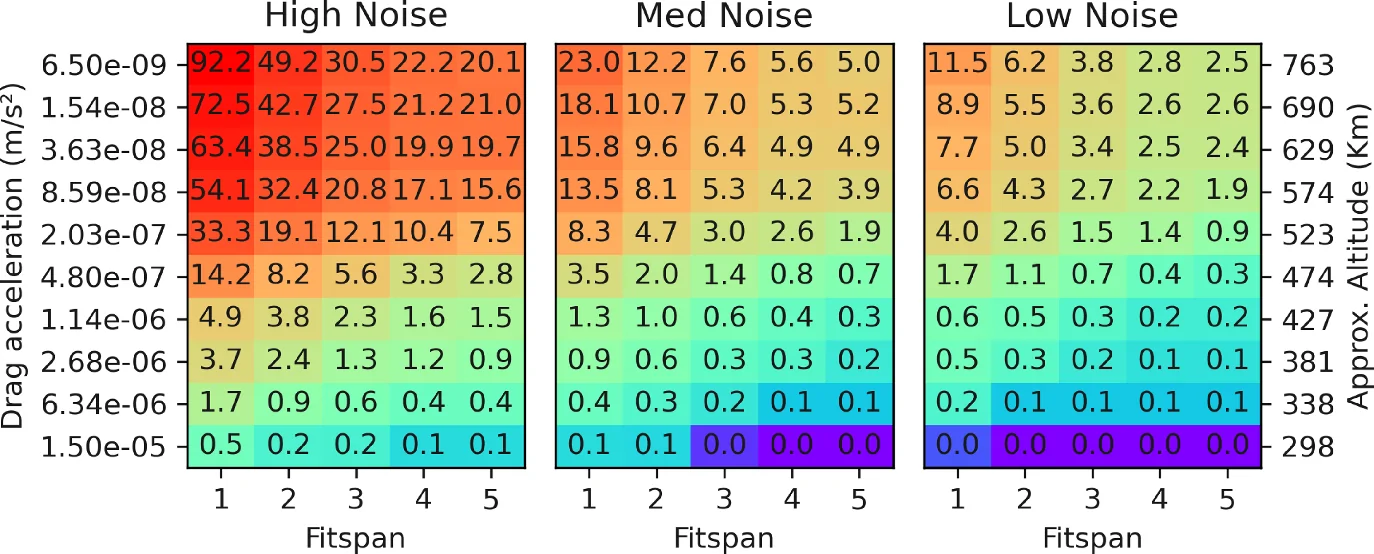
Median RMS percentage errors (Eq. 5) of densities retrieved via the EDR method (Sect. 2.2, Eq. 4) relative to HASDM, along the orbits queried over 1–7 April 2023. Each matrix corresponds to a POD noise model specified in Table 3. Values are median absolute errors across 100 orbits. Over this period (near solar maximum) the $F_{10.7}$ index ranged from 125.1 sfu to 137.6 sfu and the maximal Kp value was $K_p=4^{-}$. The secondary y-axis (right-hand side) provides approximate altitudes which correspond to the drag-accelerations on the left hand-side y-axis at the time of analysis (1-7th April 2023) for an object with $A/m=0.05\,\hbox {m}^2\,\hbox {kg}^{-1}$ and $C_D=2.2$

Figure 6 displays the absolute median error across 100 density retrievals sampling across the parameter space of interest: POD noise strength (low/medium/high as defined in table 3), fit-span length and drag acceleration. The density retrievals are carried out in 1–7 April 2023 (geomagnetically calm). The absolute median error is used here instead of the mean to reduce the impact of outliers and to give a sense of the typical error (50th percentile). Going from left to right across Fig.6, each matrix corresponds to a different noise grade (as defined in Sect. 2.3). The rows of each matrix correspond to the drag accelerations outlined in Fig. 4, and the columns corresponds to increasing fit-spans (from 1 to 5 orbital revolutions). The bottom two rows of each matrix corresponds to a very low Earth orbit region (≤350 km). In this case the strength of the drag signal is such that changes in POD quality and increases in fit-span make only marginal improvements to the quality of the retrieved densities- the signal is already far exceeding the noise of the POD solution. On the other hand, the top row in the matrices is representative of an altitude of roughly 750 km. Here, the drag signal is around four orders of magnitude weaker than for satellites in the bottom-most row, and each increment in both fit-span and POD quality, significant changes in the quality of the retrieved densities are made.

Comparing any one cell across the three matrices shows a clear ordering: the low-noise solution error exhibits roughly half the error of the medium-noise case, and the high-noise solution is typically about four times larger than the medium-noise case. This ordering and relative error magnitude is consistent with the noise levels of the POD products that these densities are derived from (see Table  3). Across all noise levels, errors decrease with increasing fit-span and with increasing drag acceleration.

We note that two of the currently operational mega-constellations (SpaceX Starlink and Amazon Kuiper- operating at around 550 and 650 km altitude respectively) operate at drag-acceleration levels where changes in fit-span, and POD noise quality stand to make a significant impact. We also note that this relationship is likely to become exacerbated over the next decade as the drag signal these constellations are exposed to will reduce in tandem with the solar cycle, which is currently exiting its maximum phase. In comparison, the frequently studied GRACE-FO platform provides ideal conditions for retrieving low error POD density estimates- especially given the well-characterized, high-quality nature of the POD data that is available for it [67].

Figure 6 shows how median RMS% errors vary with drag acceleration and fit–span for each POD–noise level. Having sampled the error space through simulation, we aim to capture these behaviours with simple functions. The aim of this exercise is to provide lightweight analytical expressions that can serve as first-order approximations of how density-retrieval error scales with drag acceleration and fit–span without the need for running a full simulation.

**Fig. 7:**
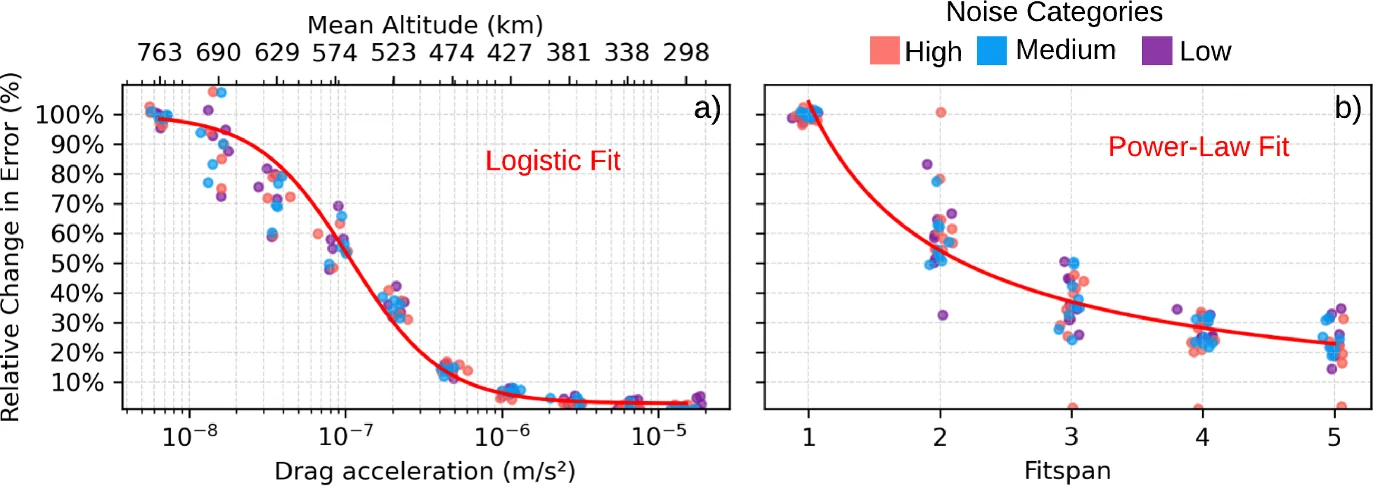
Relative change in RMS-percentage (Eq. 5) error as a function of **a** orbit-averaged drag acceleration and **b** EDR fit-span. A logistic function and a power-law provide empirical approximations for panels **a** and **b**, respectively

To construct these fits, we first normalise each row and column of the matrix by its maximum to remove absolute scale. We then analyse one axis at a time: rows for drag-dependence and columns for fit-span-dependence. These normalized values are displayed in Fig. 7. As in Fig. 6, error decreases as a function of both drag acceleration and fit-span. However there are differences in how the error decreases. Drag displays a sharper gradient in the middle of the curve, whereas fit-span sees a sharper decrease at the beginning of the curve which then tails off.

Panel (a) demonstrates that the error-drag relationship is well described by a logistic curve. At very low drag accelerations ($\sim 5\times 10^{-9}$–$2\times 10^{-8}\,\mathrm {m\,s^{-2}}$), increasing drag (done here by lowering altitude) yields only modest error reductions ($\lesssim 10\%$). Between $\sim 2\times 10^{-8}$ and $2\times 10^{-7}\,\mathrm {m\,s^{-2}}$, the curve steepens: each tripling of drag acceleration reduces the error by roughly half. Beyond $\sim 2\times 10^{-7}\,\mathrm {m\,s^{-2}}$, improvements taper as the curve approaches a floor.

This logistic response and its transition region appear consistently across all three noise levels. The noise level scales the absolute error scale (see Fig. 6). However, the relative drop in error occurs at similar drag accelerations/altitudes. We attribute this common behaviour to the orbit-averaged nature of the EDR method: across the range of noise magnitudes tested, the work done by drag over the entire orbit is consistently sufficient to produce a comparable level of improvement relative to POD noise. This behaviour mirrors the findings of [63](their Fig. 13b). Other density retrieval methods such as POD-accelerometry are reported to exhibit higher sensitivity to POD noise [63]. Sampling a broader range of noise values would clarify whether a noise dependence develops at greater noise values. Nevertheless, the current results suggest that, regardless of absolute POD noise magnitude (within the range sampled), there is a region in the drag-acceleration space where density retrieval error is disproportionately improved by a unit increase in drag acceleration.

Panel (b) shows that the fit-span dependence can be approximated by a simple power-law: the first increase in fit-span yields an approximate halving in error, and subsequent increments in fit-span have roughly half the impact of the previous increment.

Using these two mathematical fits, we can write the behaviour of the relative RMS percentage error as a separable function of drag acceleration d and fit-span f. The drag dependence in Fig.7(a) is represented by a four-parameter logistic function:

6 $$\begin{aligned} E_{\textrm{drag}}(d) = E_{\min } + \frac{E_{\max } - E_{\min }}{1 + \left( d/d_{50}\right) ^{\kappa }} \end{aligned}$$

where $E_{\max }$ is the low-drag plateau (e.g., $d \lesssim 2\times 10^{-8}\,\mathrm {m\,s^{-2}}$), $E_{\min }$ is the modelling floor (here $5\times 10^{-6}$), $d_{50}$ is the half-transition drag, and κ controls the steepness near the inflection. Over the week-long experiment, a nonlinear least-squares fit yields $E_{\max }=97\%\pm 2\%$, $E_{\min }=1.8\%\pm 0.3\%$, $d_{50}=(2.1\pm 0.2)\times 10^{-7}\,\mathrm {m\,s^{-2}}$, $\kappa =1.12\pm 0.05$, and $R^2=0.985$.

The fit-span sensitivity follows

7 $$\begin{aligned} E_{\textrm{span}}(f) = 2^{-(f-1)}, \end{aligned}$$

so that doubling the fit-span approximately halves the error.

Combining both effects gives

8 $$\begin{aligned} E(d,f) = \left[ E_{\min } + \frac{E_{\max }-E_{\min }}{1+\left( d/d_{50}\right) ^{\kappa }}\right] \times 2^{-(f-1)} \end{aligned}$$

which explains between 96% and 98% of the variance observed in the matrices for all three POD-noise levels (figure 6).

From Eqs. (6) and (7), two practical rules emerge:

- **Fit-span rule:** Each doubling of the fit-span approximately halves the RMS-percentage error, although at the cost of some temporal resolution.
- **Drag rule:** For $d \in [3\times 10^{-8},\,3\times 10^{-7}]\,\mathrm {m\,s^{-2}}$ (roughly 630-450 km), each threefold increase in d approximately halves the RMS-percentage error. Below this range, improvements are relatively minor; above it, performance approaches a floor.

These scalings were derived from the geomagnetically quiet week (1–7 April 2023, $K_p\le 4^{-}$, $F_{10.7}\approx 125\text {--}138$ sfu), circular orbits between 300 and 700 km altitude, a single orbital plane, and the three POD noise levels described in Sect. 2.3. They should be regarded as representative of moderate solar activity and quiet geomagnetic conditions; their applicability to severe storm-time conditions, significantly different solar-cycle phases, or highly eccentric orbits has not been tested and should not be assumed. The functional forms (logistic and power-law) may persist under different conditions, but the fitted coefficients ($d_{50}$, κ, $E_{\max }$, $E_{\min }$) could shift with changing solar extreme ultraviolet (EUV) flux, background density structure, and geomagnetic activity.

### Effect of Increasing Satellite Numbers and Usage Within an Assimilative Context

A central question for operational use of POD-derived densities is how much benefit can be gained by averaging independent density estimates from an increasing number of co-orbital satellites.

**Fig. 8:**
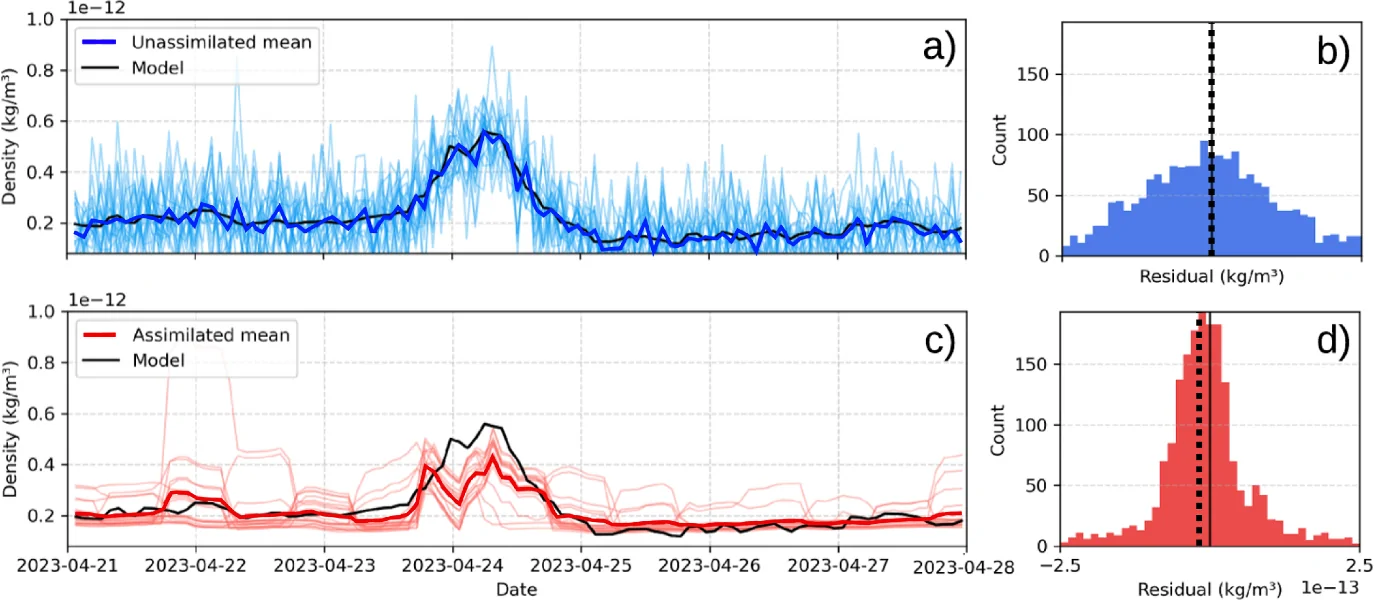
Time series of densities retrieved by 18 satellites simultaneously under the high-noise model using a fit-span of 1. Each thin blue line in panel **a** corresponds to one satellite and shows its direct density retrieval and the thick blue dotted line represents the mean of these. The thin red lines in panel **c** represent the density time series for the same satellites when processed through the assimilative pipeline. The red dotted line is the mean of those estimates. In both panels **a** and **c** the black line is the truth model (HASDM). Panels **b** and **d** show histograms of the residuals from all 18 individual satellite time series relative to the truth model with black dotted lines indicating the median

Panels a) and c) of Fig. 8 show the density time series provided by all 18 satellites along the same orbital trajectory and their mean in both processing modes: (i) direct, unassimilated density retrievals and (ii) retrievals processed through the assimilative EnKF pipeline described in Sect. 2.7. In both cases, the fit-span is fixed at 1. Individually, the assimilated time series (thin red lines in panel c) exhibit lower variance than the direct density observations (thin blue lines in panel a), as reflected by narrower residual distributions in panel d) when compared to panel b). However, when averaged, the assimilated time series exhibit bias, with a skew and offset visible in the residuals (panel d)). In contrast, individual time series in panel a) have larger per-arc errors but their mean aligns more faithfully with the density profile of the truth model (HASDM). A clear distinction emerges in the shape of the retrieved storm response by the assimilated and unassimilated models. The mean of the assimilated solutions tends toward a slightly different storm profile- characteristic of bias introduced by the underlying background model (NRLMSISE-00).

**Fig. 9:**
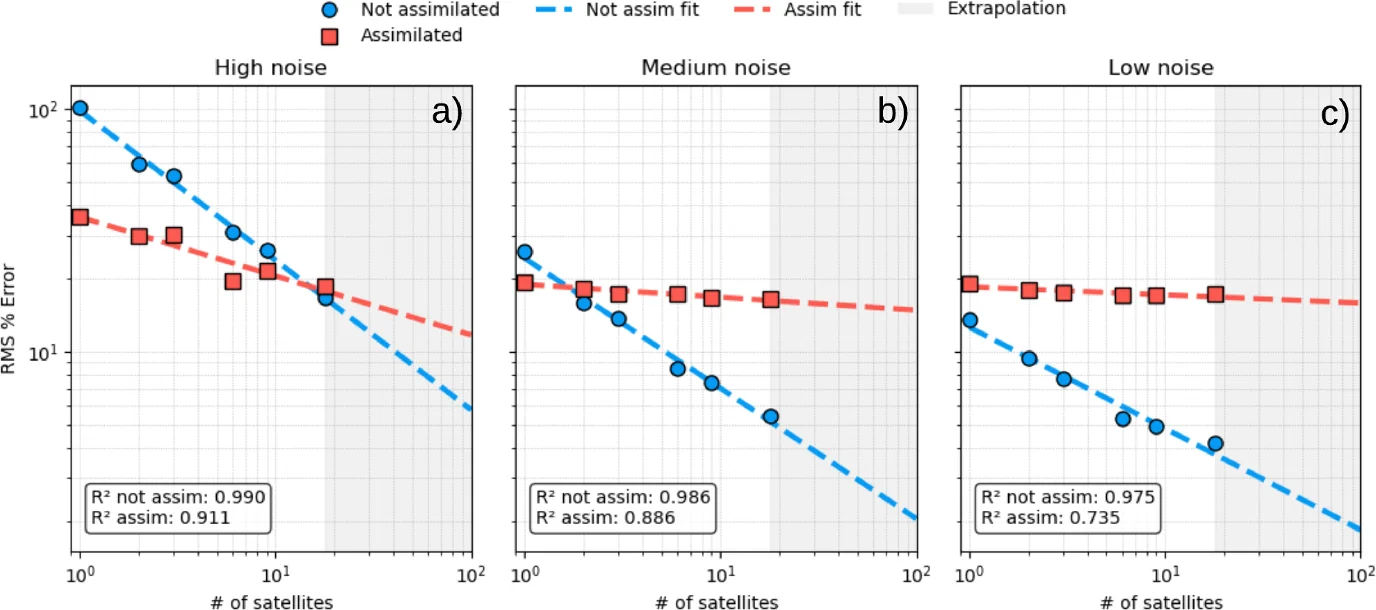
Mean RMS% error of mean-orbital density estimated by increasing numbers of equally spaced satellites. Panels **a**, **b**, and **c** correspond to high, medium and low noise levels respectively. Red line/data points are the assimilated densities. Blue line/data points are the direct density observations (unassimilated). The lines fit to the observations are inverse power-law fits. The fit lines extrapolated (grey area of graph) to 100 satellites

Furthering our effort to provide a mathematical characterisation of the drivers of POD retrieval error, we examine how error scales with the number of satellites contributing density observations. Figure 9 summarizes this relationship under the three different noise levels, both with and without assimilation. As expected, adding satellites consistently reduces error. In an ideal case of unbiased, independent, and identically distributed (i.i.d.) observations, the error should decrease according to an inverse square-root law ($\propto N^{-1/2}$). While this scaling provides a useful first-order approximation, our results are more accurately described by an inverse power law with a shallower slope. We believe this departure from the theoretical $\sim N^{-1/2}$ behaviour arises from the structure of the proposed noise model, which introduces autocorrelation, bias, and common error modulation across satellites (i.e. through GDOP effects), thereby breaking the i.i.d. assumption.

Both the absolute value of the error (intercept), and to a lesser extent the gradient of the slope, are modulated by POD noise level and whether assimilation is used. Under the high POD noise case, a given increase in the number of satellites used yields a stronger improvement for the unassimilated case. When densities are generated via the assimilative tool, the incremental gain from additional satellites is smaller.

A key finding of this study is the crossover visible in Fig. 9, where the blue (unassimilated) curves fall below the red (assimilated) curves. Beyond this satellite-count threshold, the simple mean of unassimilated retrievals outperforms the mean of individually assimilated estimates in resolving densities along the arc. The mechanism is as follows: each per-satellite assimilation maps the retrieved density onto the NRLMSISE-00 background model by adjusting $A_p$. Any systematic bias in NRLMSISE-00 (e.g. an incorrect storm-time density profile) is thereby imprinted on every assimilated time series. This is a general property of data assimilation: a biased background model invariably produces a biased analysis [19]. Because the bias is common to all satellites, averaging more assimilated series does not cancel it; it persists. By contrast, the unassimilated retrievals are independent of the background model; their errors are dominated by POD noise, which does cancel under averaging. Once sufficiently many satellites contribute, the noise-averaging benefit of the unassimilated mean exceeds the variance-reduction benefit of assimilation, and the unassimilated mean becomes more accurate along the sampled orbit.

We stress that this does not imply that assimilation lacks value. The RMS% errors reported in Fig. 9 quantify performance along the specific orbital arc. Assimilation provides a global density field, whereas the unassimilated mean is constrained to the sampled orbit. Hence, while the unassimilated mean can achieve lower error along this specific orbit given enough satellites, its utility is inherently more limited in scope.

We find a good approximation of the relationship between the error E(N) and the number of satellites N is provided by the power-law rule:

9 $$\begin{aligned} \frac{E(N)}{E(1)} \;\approx \; N^{-b} \quad \Longrightarrow \quad E(N)\;\approx \;E(1)\,N^{-b} \end{aligned}$$

Fitting across all POD tiers yields

$$ b \;\approx \; {\left\{ \begin{array}{ll} 0.6 &  \text {(unassimilated)}\\ 0.25 &  \text {(assimilated)} \end{array}\right. } $$

The RMS% error of the direct (unassimilated) observations decays approximately as$E(N)\approx E(1)\,N^{-0.6}$, so doubling the satellite count reduces error by $2^{-0.6}\approx 0.6598$ (a 34.0% reduction). In the assimilative context, the decay is shallower, $E(N)\approx E(1)\,N^{-0.25}$, so doubling the satellite count reduces error by $2^{-0.25}\approx 0.8409$ (a 15.9% reduction). Goodness-of-fit for the full three-parameter approximation is reported below.

### An Analytical Approximation of Density Inversion Performance

To incorporate the effect of increasing the number of satellites N into our mathematical model, we combine Eq. 9 with Eq. 8. The full three-parameter error surface is given by

10 $$\begin{aligned} E(d,f,N)\;=\;E_{\min }\;+\;\frac{E_{\max }-E_{\min }}{1+(d/d_{50})^{\kappa }}\;2^{-(f-1)}\,N^{-b} \end{aligned}$$

where E is the RMS percentage error (Eq. 5), d is the orbit-averaged drag acceleration $(\hbox {m s}^{-2})$, f is the fit-span (in number of orbits), and N is the satellite count. The exponents κ and b are dimensionless; b≈0.6 for unassimilated retrievals and b≈0.25 for assimilated retrievals.

Relative to the cell-wise RMS% errors in Fig. 6, the full analytical model (Eq.10) captures around 89% of the variation in the simulation data. Expressed as the coefficient of determination ($r^2$), which measures the fraction of variance explained by the model, the values are $r^2_{\text {high}}=0.894$, $r^2_{\text {medium}}=0.892$, and $r^2_{\text {low}}=0.887$, corresponding to the high-, medium-, and low-noise tiers respectively.

Equation 10 assumes that the effects of drag, fit-span, and satellite count are separable (i.e. multiplicative). We tested all three pairwise interactions by fitting the relevant exponent (fit-span decay or satellite-count averaging) at each level of the other factor. In all cases the exponents were stable: the fit-span decay exponent did not vary systematically with drag level (coefficient of variation <10%), and the satellite-count averaging exponent did not vary with either drag level or fit-span (coefficient of variation $\sim \!10\%$). This separability is physically expected: drag governs the signal-to-noise ratio of the energy dissipated along each arc, fit-span reduces variance by averaging successive orbit-mean observations, and satellite count reduces variance by averaging co-orbital estimates at each epoch. Because these three mechanisms operate on distinct aspects of the retrieval, their effects combine multiplicatively.

## Discussion

In the preceding section we found that retrieval error decreases approximately as $2^{-(f-1)}$ with fit-span, follows a logistic–like response to drag with the steepest section of the logistic curve between $2\times 10^{-8}$ and $2\times 10^{-7}\,\mathrm {m\,s^{-2}}$, and declines with satellite count as $N^{-0.6}$ (unassimilated) or $N^{-0.25}$ (assimilated).

The halving in density-retrieval error with increasing fit–span (Eq. 7) is consistent with variance reduction by averaging and persists in the presence of coloured, anisotropic, and autocorrelated POD errors (Sect. 2.3). This indicates that, across independent orbit-averaged density observations of one same spacecraft, the dominant error structures in the modelled LEO POD noise, exhibit sufficiently short temporal correlation that successive arcs contribute quasi-independent information. This is plausible given that LEO satellites complete one revolution in approximately 90 min, during which the Earth rotates roughly $22.5^{\circ }$; successive orbits therefore sample different longitude bands, reducing spatial correlation between consecutive arc-averaged densities. The benefit of increasing the fit–span must, however, be balanced against the temporal scale of the density signal one wishes to resolve: certain features of a storm can, in certain cases, materialise on time scales below that of a single orbit (≳100 minutes) [56, 76]. Under such conditions, using large fit–spans risks smoothing signals of operational interest. In a modelling context, the fit–span should therefore be selected to preserve the temporal scales of interest while reducing retrieval variance, rather than to minimise variance alone.

The relationship between retrieval error and drag acceleration magnitude is well described by a logistic curve (Fig. 7a; Eq. 6), with a low-drag plateau, a transition region in which each $\sim 3\times$ increase in drag acceleration reduces error by approximately one half, and a high-drag floor with diminishing returns. In our experiments, this steep region lies between $2\times 10^{-8}$ and $3\times 10^{-7}\,\mathrm {m\,s^{-2}}$.

The high-drag floor primarily reflects effects intrinsic to the EDR method, and thus still persist even in a fully simulated, internally consistent setup. First, by construction $\rho _{\textrm{eff}}$ is a weighted mean of $C_D A v_{\textrm{rel}}^{3}$ (see Eq. 4), whereas our reference "truth" is the simple mean of HASDM densities along the orbit. These coincide perfectly only if the weight $C_D A v_{\textrm{rel}}^{3}$ is constant or statistically independent of ρ; in practice $v_{\textrm{rel}}$ varies along the arc and ρ varies with latitude and local time, so a small bias between the two averages remains even as random noise vanishes. Second, finite perigee-to-perigee segmentation and discrete sampling of the orbital energy E(t) and numerical quadrature of the denominator in equation 4 introduce small, drag-independent biases. As drag increases and random error becomes negligible, this methodological floor dominates, producing the observed saturation.

Because the trajectories and force model are internally consistent in our simulations, residual non-drag mismodelling should not contribute; however, in operational POD data such effects can matter [62]. While precise thresholds are expected to vary with satellite geometry, area-to-mass ratio, attitude law, and the chosen reference (“truth”) density model, the overall shape of the relation is consistent with prior studies on the sensitivity of EDR to POD noise [63]. Although spacecraft operators typically aim to minimise the effects of drag on their spacecraft, the results presented here indicate that in some cases it may be advantageous to deliberately increase the drag experienced by a subset of platforms within a constellation. This could be achieved, for example, by maintaining a slightly lower orbital altitude or by temporarily increasing the projected area into the atmospheric flow. A stronger drag signal enhances the accuracy of density retrievals, and this in turn improves the quality of the underlying assimilative model. Better density characterization will propagate into reduced state uncertainties and orbit propagation errors. In turn, these can reduce manoeuvre frequency, for instance by improving the reliability of conjunction probability assessments [1, 11, 12].

Constellations typically provide multiple satellites distributed along a common orbital track. Exploiting this geometry offers a third lever via platform count, an effect that has been less explored in the literature. For direct (unassimilated) means of simultaneous density observations, we find that the error drops according to the relation: $E(N)\sim N^{-0.6}$ (Fig. 9), which is slightly steeper than the $N^{-1/2}$ reduction expected of independent, identically distributed errors. This behaviour may be suggestive of partial phase cancellation: error components with systematic orbital-phase structure (i.e. driven by geometry or GDOP modulation) tend to have opposite signs at different phase offsets; equal spacing of satellites therefore cancels part of this structured component when averaging, in addition to reducing random variance (see Sect. 2.3). In contrast, averaging individually *assimilated* estimates yields a much shallower $N^{-0.25}$ decay in error as satellites are added. In the results presented, this is likely indicative of background-model bias leakage. When assimilation is performed per-satellite (method outlined in Sect. 2.7), common biases in the background density model are not removed and are re-introduced in each density time series, so averaging more assimilated series does not cancel the shared component. Expanding the scheme to include simultaneous assimilation of multiple satellite data streams of various orbital geometries is expected to recover a steeper N-scaling than the per-satellite baseline presented here. The exponent b≈0.25 (in Eq. 9) should therefore be regarded as a lower bound for assimilated pipelines that do not explicitly address common error sources.

The assimilated results may also be influenced by the limited spatial sampling of a single orbital plane during storm conditions. The multi-satellite analysis in Sect. 3.3 was performed during the G4 storm of 21–28 April 2023 ($K_p$ up to $8^+$), where rapid spatial and temporal density gradients challenge any single-plane configuration. It is possible that the specific orbital geometry used (Table 1) was not optimally placed to resolve these storm-time density variations, contributing to the relatively shallow improvement with satellite count seen in the assimilated case.

A practical finding for current operators is that, along a well-sampled orbital path, the simple arithmetic mean of several unassimilated EDR-based density retrievals can provide highly accurate along-track density estimates (depending on POD noise and number of satellites used). This does not diminish the role of assimilation, which remains essential for producing a coherent four-dimensional density field and for propagating information to unsampled regions [22, 68, 69]. Rather, it identifies useful cases in which assimilation may not even be necessary. Firstly, if the objective is simply the specification of density along a single orbital track, direct averaging can provide high quality density information at relatively low computational cost and technical complexity. Secondly, this "string-of-pearls" configuration also enables effective anomaly detection and outlier removal by comparing each satellite to its in-plane peers. Anomalies could for example be the result of unanticipated thruster or attitude maneouvers or a bad onboard GNSS solution.

Taken together, the fitted relationships (Eq. 10) provide a practical first-order mapping from performance requirements to design choices. For large constellations, even a modest, well-distributed subset of higher-quality POD solutions should be able to deliver density estimates approaching single-digit RMS% error under favourable conditions when coupled appropriately with other processing parameters. The power-law extrapolation shown in Fig. 9 suggests that this may be achievable, but such extrapolation should be treated with caution. At sufficiently large satellite counts, systematic error sources common to all satellites, including force-model biases, $C_D$ uncertainty, and shared GDOP structure, are expected to impose an error floor below which additional satellites yield diminishing returns. Achieving the lowest extrapolated error levels would require addressing these systematic sources rather than simply increasing N.

Several assumptions bound the applicability of these results. Using HASDM as the reference density model likely influences the error floor and the apparent logistic inflection (Fig.7); repeating the analysis with alternative reference density models (e.g., NRLMSISE-00, JB2008, WAM-IPE) will shift thresholds but is unlikely to alter the qualitative relationships described. Increasing the physical fidelity of the drag model (time-varying area and $C_D$, realistic attitude law) will introduce additional variability, primarily through modulation of the drag signal [27]. While the noise model used in this study advances beyond a white-noise assumption, it does not include all operational non-stationarities (e.g. receiver duty cycles, clock events). Moreover, absolute noise levels were drawn from a limited set of literature sources because independent validation of commercial-class POD remains scarce.

An important caveat concerns the generalisability of the assimilation results. Because NRLMSISE-00 is an empirical model whose density at time *t* does not depend on density at time t-1, the scheme presented here is limited to estimating corrections to model inputs rather than updating atmospheric state. In General Circulation Models, updated state variables influence future predictions through resolved dynamics, and standard data assimilation techniques (e.g. 4DVar, EnKF applied to state vectors) can propagate observational information forward in time. The scaling relationships derived here, particularly the satellite-count exponents and the assimilation-related findings, are specific to empirical-model calibration and should not be assumed to transfer directly to GCM-based assimilation systems. The fit-span and drag-signal scalings, which characterise the raw retrieval step prior to assimilation, are expected to be more broadly applicable.

Further advances in this line of work are likely to be limited by the lack of public characterisation of commercial-class LEO POD quality [4, 66]. Reliable assessments of density retrieval method performance depend on credible, independently validated error budgets. Science missions routinely employ SLR for this purpose [36, 48]; equipping even a small number of spacecraft within large constellations with SLR retro-reflectors, would materially strengthen the certainty and quality of density retrievals and accelerate improvements in processing and assimilation. Wider dissemination of low-latency LEO POD data (both commercial and scientific), where licensing permits, would also further advance work in this field.

## Conclusions

This study presents a sensitivity analysis of the key parameters governing density retrieval performance when the EDR method is applied to commercial-grade POD data for use in assimilative models. To our knowledge, no such systematic characterisation exists in the literature, leaving model developers without a quantitative basis for specifying POD quality requirements or constellation sampling strategies. We find that retrieval error can be approximated by three rules of thumb relating to fit-span, drag-acceleration level, and satellite count—relationships intended to support design-to-requirement mapping for next-generation assimilative systems. Additionally, we show that the commonly adopted white-noise assumption may overestimate retrieval errors, suggesting that existing performance assessments based on white noise may be overly pessimistic.

By replacing an isotropic white-noise surrogate with a literature-informed higher-fidelity POD error model (anisotropy, pink along-track colouring, short-range autocorrelation, GDOP-modulated heteroscedasticity, and a small along-track bias) and by assessing a per-satellite EnKF that adjusts $A_p$ to a background model (NRLMSISE-00), we recover analytical scalings which aim to support the design of next-generation assimilative pipelines.

First, the fit-span lever behaves as expected from variance-reduction principles but remains effective even under realistic error models: every doubling of the EDR fit–span reduces RMS-percentage error by approximately one half. This scaling persisted across the noise levels tested, indicating that successive orbit-mean observations contain sufficiently independent information even when along-track POD errors are somewhat autocorrelated. The practical consequence for model designers is not to maximise fit–span indiscriminately, but to select the shortest span which still preserves storm-time variability of interest while still meeting accuracy targets; over-smoothing forfeits the temporal resolution of signals that matter operationally.

Second, the drag-signal lever follows a logistic-like response. At very low drag accelerations ($\lesssim 2\times 10^{-8}\,\mathrm {m\,s^{-2}}$) increasing the drag signal yields only marginal improvement. Across a transition band of approximately $2\times 10^{-8}$ to $2\times 10^{-7}\,\mathrm {m\,s^{-2}}$, each $\sim \!3\times$ increase in drag delivers a $\sim \!50\%$ reduction in error. Beyond $\gtrsim 10^{-6}\,\mathrm {m\,s^{-2}}$ the curve approaches a floor, reflecting diminishing returns.

Many operational and planned satellite constellations span this transition region (450–650 km); modest changes in operating altitude or in the effective projected area of the satellite (e.g. via changes in attitude) can be used to tune the strength of the drag acceleration and achieve density retrieval accuracy improvements. In practice, however, both levers are tightly constrained by primary mission requirements (e.g. lifetime, coverage, pointing for power/communications), so density retrieval is likely to remain a secondary objective. Nevertheless, awareness of these scalings enables opportunistic drag tuning: operators may exploit existing attitude modes or scheduled operations that transiently change projected area (e.g. downlink pointing, momentum dumps, sun-pointing/charging attitudes) or natural altitude dispersions within their broader constellation to harvest higher signal-to-noise EDR observations without the need for dedicated manoeuvres.

Thirdly, increasing the number of simultaneously used satellites provides an effective lever. When density estimates from N equally spaced co-orbital satellites are combined, the direct (unassimilated) mean of those densities follows $E(N)\approx E(1)\,N^{-0.6}$: errors fall faster than the idealised $N^{-1/2}$ expectation associated with random noise. We suggest this may be due to partial cancellation of phase-structured components (e.g. GDOP-modulated signatures) in addition to reduction of random variance arising from POD noise. When each satellite’s time series is first assimilated independently and the results are then averaged, the improvement is shallower, $E(N)\approx E(1)\,N^{-0.25}$. In our configuration this behaviour is attributable to common background-model bias that persists across per-satellite runs and thus does not cancel under averaging. The implication is twofold. Along a well-sampled orbital path, a simple arithmetic mean of multiple unassimilated EDR retrievals can even exceed the average of individually assimilated series, provided the POD noise is low enough and N is sufficiently large. At the same time, assimilation remains essential for producing a coherent four-dimensional density field and propagating information away from sampled tracks; a joint, multi-satellite assimilation is expected to mitigate shared-bias effects and recover steeper N-scaling than the per-satellite baseline assessed here.

The fidelity with which POD errors are characterized and then modelled matters. For an equivalent absolute 3D RMS error level, the higher-fidelity noise model produced smaller density-retrieval errors than a white-noise approach. Simplistic noise models may channel error through pathways that are unrepresentative of operational POD products. This result motivates the need for further LEO POD data gathering and characterization efforts, especially of the kinds that are used by commercial satellite operators.

The observed behaviours can be summarized in an analytical form that explains the bulk of the observed variance ($\ge 89\%$) and is provided in the aim to support design-to-requirement mapping:

$$ E(d,f,N)\;=\;E_{\min }\;+\;\frac{E_{\max }-E_{\min }}{1+\left( d/d_{50}\right) ^{\kappa }}\;2^{-(f-1)}\,N^{-b} $$

where d is orbit-averaged drag acceleration, f the fit–span (in orbits), and N the in-plane satellite count, with $b\!\approx \!0.6$ for direct averaging and $b\!\approx \!0.25$ for the per-satellite assimilation used here. This analytical approximation is able to capture ∼89% of the variance in the simulated data.

With the exception of the assimilated satellite-count scaling exponent, these results relate the performance of commercial-grade POD products to the accuracy of derived densities, and are pertinent to the ingestion of EDR-derived observations into any assimilative empirical model. The error scaling relationships may serve as an initial basis for specifying observation error covariances, data-screening thresholds, and processing strategies in operational systems.

## Future Work

The orbit-local $A_p$ correction estimated by the per-satellite calibration scheme improves NRLMSISE-00 density predictions along the sampled orbital arc, but may degrade predictions at other locations (different local times, latitudes, and altitudes) where the correction is not representative. Characterising this spatial trade-off is an important direction for future work.

Further work would also benefit from incorporating simultaneous multi-satellite assimilation, higher-fidelity force modelling (time-varying area and $C_D$), investigating the use of adaptive fit-spans, and a broader range of orbital geometries. We also plan to further validate the results presented herein against real POD data arcs and independent truth sources (e.g. accelerometer-derived densities), developing the results provided into a more detailed analysis which could be used as a document to support assimilative model development by enabling developers to quantitatively specify requirements (e.g. POD quality) and processing strategies for the next generation of assimilative empirical thermospheric density models.

## Data Availability

The simulation datasets generated and analysed during this study are available from the corresponding author upon reasonable request. No external or third-party datasets were used.
